# Development and validation of prognostic nomograms based on De Ritis ratio and clinicopathological features for patients with stage II/III colorectal cancer

**DOI:** 10.1186/s12885-023-11125-5

**Published:** 2023-07-03

**Authors:** Jinming Fu, Fenqi Du, Tian Tian, Hao Huang, Lei Zhang, Dapeng Li, Yupeng Liu, Ding Zhang, Lijing Gao, Ting Zheng, Yanlong Liu, Yashuang Zhao

**Affiliations:** 1 Department of Epidemiology, College of Public Health, Harbin Medical University, 157 Baojian Road, Harbin, 150081 Heilongjiang Province People’s Republic of China; 2 Department of Biostatistics, School of Public Health, Xuzhou Medical University, Xuzhou, 221004 China; 3 Department of Colorectal Surgery, Harbin Medical University Cancer Hospital, Harbin Medical University, 150 Haping Road, Harbin, 150081 Heilongjiang Province People’s Republic of China

**Keywords:** Colorectal cancer, Prognosis, Preoperative serum liver enzyme markers, De Ritis ratio, Nomograms

## Abstract

**Background:**

Metabolic derangements and systemic inflammation are related to the progression of colorectal cancer (CRC) and the prognoses of these patients. The survival of stage II and III CRC patients existed considerable heterogeneity highlighting the urgent need for new prediction models. This study aimed to develop and validate prognostic nomograms based on preoperative serum liver enzyme as well as evaluate the clinical utility.

**Methods:**

A total of 4014 stage II/III primary CRC patients pathologically diagnosed from January 2007 to December 2013 were included in this study. These patients were randomly divided into a training set (*n* = 2409) and a testing set (*n* = 1605). Univariate and multivariate Cox analyses were used to select the independent factors for predicting overall survival (OS) and disease-free survival (DFS) of stage II/III CRC patients. Next, nomograms were constructed and validated to predict the OS and DFS of individual CRC patients. The clinical utility of nomograms, tumor-node-metastasis (TNM), and the American Joint Committee on Cancer (AJCC) system was evaluated using time-dependent ROC and decision curve analyses.

**Results:**

Among seven preoperative serum liver enzyme markers, aspartate aminotransferase-to-alanine aminotransferase ratio (De Ritis ratio) was identified as an independent factor for predicting both OS and DFS of stage II/III CRC patients. The nomograms incorporated De Ritis ratio and significant clinicopathological features achieved good accuracy in terms of OS and DFS prediction, with C-index of 0.715 and 0.692, respectively. The calibration curve showed good agreement between prediction by nomogram and actual observation. The results of time-dependent ROC and decision curve analyses suggested that the nomograms had improved discrimination and greater clinical benefits compared with TNM and AJCC staging.

**Conclusions:**

De Ritis ratio was an independent predictor in predicting both the OS and DFS of patients with stage II/III CRC. Nomograms based on De Ritis ratio and clinicopathological features showed better clinical utility, which is expected to help clinicians develop appropriate individual treatment strategies for patients with stage II /III CRC.

**Supplementary Information:**

The online version contains supplementary material available at 10.1186/s12885-023-11125-5.

## Background

Globally, colorectal cancer (CRC) is the second leading cause of cancer death, accounting for an estimated 915,880 deaths in 2020 [1]. Surgical resection is the main radical treatment for CRC, however, approximately one-half of patients recurred within the first 3 years after surgery [2, 3]. Despite tumor, node, metastasis (TNM) staging at the time of diagnosis is an important basis to distinguish the survival of CRC patients, the heterogeneity of prognosis also existed in patients with the same TNM stage [4, 5]. Providing individual recurrence/metastasis probabilities prediction for CRC patients, and adapting the treatment and follow-up frequency accordingly can improve the survival of patients. Therefore, it highlights the urgent need for developing new prognosis prediction models.

Metabolic reprogramming is a hallmark of cancer. Accumulating evidence suggests that metabolic derangements provide abundant energy, nutrients, and redox requirements for tumor cells, which contributes to the occurrence and progression of tumor [6]. Serum biomarkers derived from clinical routine testing are being widely used in the diagnosis, follow-up, and prognosis of tumors because they are non-invasive, economical, and easy to measure. Abnormal levels of serum liver enzyme markers, such as lactate dehydrogenase, alkaline phosphatase, and aspartate aminotransferase-to-alanine aminotransferase ratio (De Ritis ratio), could indicate metabolic derangements in the tumor microenvironment. The potential ability of these markers for predicting the prognosis has been evaluated in several cancers [7–14]. With the discovery that inflammatory cells actively participate in tumor progression [15], serum markers combining serum liver enzymes with peripheral blood cells were also constructed to predict the prognosis of patients with hepatocellular carcinoma, intrahepatic cholangiocarcinoma, and metastatic CRC [16–19]. However, the role of serum liver enzyme markers in non-metastatic CRC is still unclear.

Therefore, our study evaluated the prognostic values of seven preoperative serum liver enzyme markers. Considering the ability of a single serum biomarker may be insufficient, we incorporated significant serum liver enzyme markers and clinicopathological features to develop prognostic nomograms for a better individual CRC patient’s survival prediction.

## Methods

### Study population

**Fig. 1:**
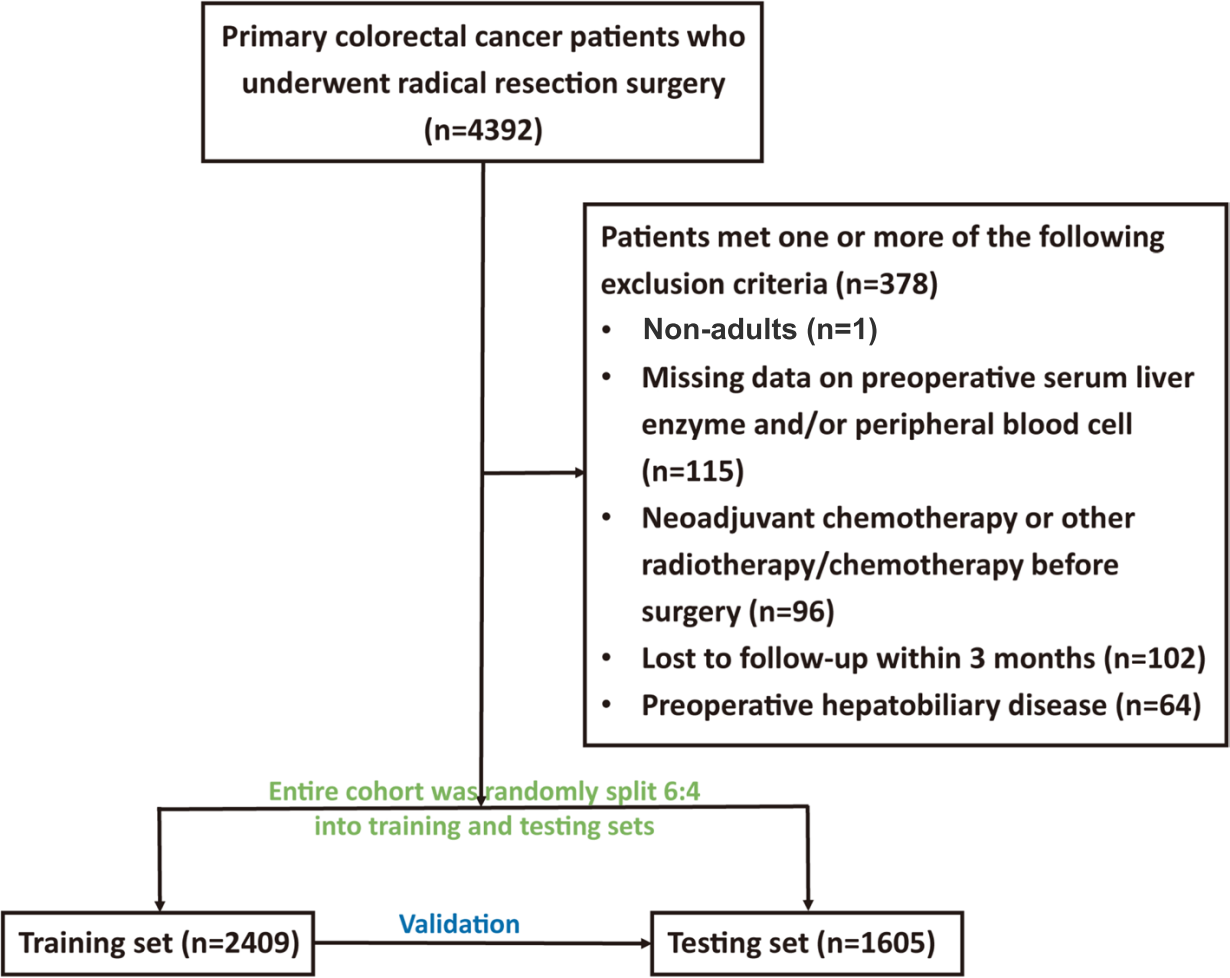
Detailed flow chart of patient selection in this study

A total of 4392 primary stage II/III CRC patients confirmed by pathological diagnosis were enrolled in this retrospective cohort. These patients underwent radical resection surgery in the Third Affiliated Hospital of Harbin Medical University from January 2007 to December 2013. Patients who met one or more of the following exclusion criteria were excluded (Fig. 1): non-adults (*n* = 1); missing data on preoperative serum liver enzyme and/or peripheral blood cell (*n* = 115); patients received neoadjuvant chemotherapy or other radiotherapy/chemotherapy before surgery (*n* = 96); patients lost to follow-up within 3 months (*n* = 102); and patients with hepatobiliary disorders (*n* = 64).

At last, 4014 stage II/III CRC patients were included in this study and these patients were randomly divided into a training set (60%) and a testing set (40%). Throughout this article, the term ‘‘prognostic marker’’ is defined according to REMARK Guidelines [20].

### Data collection

Patients’ demographic and clinicopathological features were obtained from retrospective medical records. The pathological staging of patients was defined using both the traditional TNM staging and the American Joint Committee on Cancer (AJCC) staging, respectively. Data on lactate dehydrogenase, alkaline phosphatase, aspartate aminotransferase, alanine aminotransferase, platelet, lymphocyte, and neutrophil were extracted from the results of the first blood routine tests and biochemical tests (limit to 30 days prior to surgery). Blood routine tests and biochemical tests were based on a single blood sample of each patient and were measured by auto analyzers.

The De Ritis ratio, aspartate aminotransferase-to-platelet ratio index (APRI), aspartate aminotransferase-to-lymphocyte ratio index (ALRI), aspartate aminotransferase-to-neutrophil ratio index (ANRI), and alkaline phosphatase-to-platelet ratio index (APPRI) were calculated using the following formulas: De Ritis ratio = aspartate aminotransferase level (U/L)/alanine aminotransferase level (U/L) [21]; APRI = (aspartate aminotransferase level (U/L)/platelet counts (10^9^/L)) × 10^9^/U [16]; ALRI = (aspartate aminotransferase level (U/L)/lymphocyte counts (10^9^/L)) × 10^9^/U [17]; ANRI = (aspartate aminotransferase level (U/L)/neutrophil counts (10^9^/L)) × 10^9^/U [18]; APPRI = (alkaline phosphatase level (U/L)/platelets counts (10^9^/L)) × 10^9^/U [19].

Patients were followed up regularly according to NCCN guidelines. The last time of follow-up was January 22, 2019. The survival information was obtained from contacts with patients by phone. Overall survival (OS) was defined as the period from surgery to death from any cause, or the last contact. Disease-free survival (DFS) was defined as the period from surgery to local recurrence, distant metastasis, a new primary tumor of CRC, or death, whichever comes first.

### Statistical analysis

Multiple imputation was conducted to fill the missing data of the included variables [22]. Student’s *t* tests for normally distributed continuous variables, *χ*^*2*^ tests for categorical variables, and Mann–Whitney *U* tests for non-normally distributed continuous variables were performed to evaluate the differences between training and testing sets. X-tile 3.6.1 software (Yale University, New Haven, CT, USA) [23] was used to determine the optimal cut-off values for lactate dehydrogenase, alkaline phosphatase, De Ritis ratio, APRI, ALRI, ANRI, and APPRI. Additionally, the associations between these serum liver enzyme markers and clinicopathological features were explored by using *χ*^*2*^ tests.

The 1-, 3-, and 5-year OS and DFS were calculated using the Kaplan–Meier method, and the survival differences of CRC patients between low and high levels of serum liver enzymes were compared using log-rank tests. The prognostic values of clinicopathological features and preoperative serum liver enzyme markers were estimated using univariate and multivariate Cox proportional hazards models, and the results were presented as hazard ratio (HR) and 95% confidence interval (CI). Subgroup analyses were also conducted stratified by age, gender, tumor location, tumor diameter, CEA, and CA19-9.

The nomograms that combined significant serum liver enzyme markers and clinicopathological features were developed, to predict the probability of 1-, 3- and 5-year survival recurrence/metastasis of patients with stage II/III CRC in the training and testing sets. The variables with a *P* < 0.05 in the multivariate Cox analyses were identified as significant prognostic factors and were finally incorporated into the nomograms. Nomograms map the predicted probabilities into points on a scale from 0 to 100 and can be interpreted by accumulating the points corresponding to the predicted probability, which is indicated at the top of the scale [24–26].

The prediction accuracy of nomograms was evaluated by the concordance index (C-index) [27]. Bootstrapping techniques were used for internal validation of the prognostic models, and the calibration of nomograms was assessed by plotting the actual probabilities versus the nomogram predicted probabilities [27]. Time-dependent ROC analyses were performed, and the estimated AUCs were calculated to compare the discriminative ability of the nomograms, TNM, and AJCC staging [28, 29]. Finally, decision curve analyses were performed to evaluate the net benefits of nomograms, TNM, and AJCC staging under different threshold probabilities, thereby comparing the clinical utility of these three models [30–32].

All the statistical analyses were conducted with SPSS 24.0 (SPSS Inc., Chicago, IL, USA) and R 4.1.2 software (Institute for Statistics and Mathematics, Vienna, Austria). Two-sided *P* < 0.05 was considered statistically significant.

## Results

### Clinical characteristics of patients in the training and testing sets

Overall, 2409 stage II and 1605 stage III CRC patients were included in this study. The mean age in the training and testing sets were 59.4 and 59.0, respectively. The median follow-up time was 71.0 months (interquartile ranges: 50.0–94.0 months) in the training set, with 748 deaths during this period. The median follow-up time was 69.0 months (interquartile ranges: 49.0–92.0) in the testing set, with 476 deaths during this period. Details of the clinical characteristics of the two sets are summarized in Table 1. No significant difference was observed in terms of the clinical characteristics between the training and testing sets (all *P* > 0.05).

**Table 1:** Baseline characteristics of patients in the training and testing sets

Characteristics	Category	Training set (*n* = 2409)	Testing set (*n* = 1605)	*P* value
**Age (year) **^**a,b**^	/	59.4 (11.69)	59.0 (11.55)	0.368
	< 60	1219 (50.6)	826 (51.5)	0.615
	≥ 60	1190 (49.4)	779 (48.5)	
**Gender **^**b**^	Male	1436 (59.6)	945 (58.9)	0.668
	Female	973 (40.4)	660 (41.1)	
**BMI (kg/m2) **^**b**^	< 24	1401 (58.2)	915 (57.0)	0.517
	≥ 24	1008 (41.8)	690 (43.0)	
**Tumor location **^**b**^	Colon	1140 (47.3)	751 (46.8)	0.766
	Rectum	1269 (52.7)	854 (53.2)	
**Tumor diameter **^**b**^	< 50 mm	968 (40.2)	681 (42.4)	0.152
	≥ 50 mm	1441 (59.8)	924 (57.6)	
**Pathological classification **^**b**^	Prominence	1570 (65.2)	1080 (67.3)	0.379
	Infiltration or ulceration	253 (10.5)	160 (10.0)	
	Infiltration and ulceration	586 (24.3)	365 (22.7)	
**Differentiation degree **^**b**^	Well	189 (7.8)	130 (8.1)	0.630
	Moderate	1861 (77.3)	1253 (78.1)	
	Poor	359 (14.9)	222 (13.8)	
**Histologic classification **^**b**^	Adenocarcinoma	1819 (75.5)	1213 (75.6)	0.991
	Mucinous adenocarcinoma + signet ring cell carcinoma	590 (24.5)	392 (24.4)	
**TNM staging **^**b**^	II	1384 (57.5)	922 (57.4)	1.000
	III	1025 (42.5)	683 (42.6)	
**AJCC staging II **^**b**^	IIA	567 (41.0)	379 (41.1)	0.357
	IIB	94 (6.8)	78 (8.5)	
	IIC	723 (52.2)	465 (50.4)	
**AJCC staging III **^**b**^	IIIA	70 (6.8)	52 (7.6)	0.354
	IIIB	423 (41.3)	306 (44.8)	
	IIIC	532 (51.9)	325 (47.6)	
**Tumor invasion **^**b**^	T1-T3	1061 (44.0)	733 (45.7)	0.326
	T4	1348 (56.0)	872 (54.3)	
**Lymph nodes involved **^**b**^	N0	1384 (57.5)	922 (57.4)	1.000
	N1-N2	1025 (42.5)	683 (42.6)	
**Tumor deposits **^**b**^	No	2250 (93.4)	1482 (92.3)	0.219
	Yes	159 (6.6)	123 (7.7)	
**CEA **^**b**^	< 5 ng/mL	1417 (58.8)	936 (58.3)	0.830
	≥ 5 ng/mL	992 (41.2)	669 (41.7)	
**CA19-9 **^**b**^	< 37U/mL	2020 (83.9)	1347 (83.9)	0.872
	≥ 37U/mL	389 (16.1)	258 (16.1)	
**Postoperative chemotherapy **^**b**^	No	1416 (58.8)	908 (56.6)	0.176
	Yes	993 (41.2)	697 (43.4)	
**Postoperative radiotherapy **^**b**^	No	2296 (95.3)	1541 (96.0)	0.325
	Yes	113 (4.7)	64 (4.0)	
**HBs-Ag **^**b**^	Negative	2294 (95.2)	1519 (94.6)	0.449
	Positive	115 (4.8)	86 (5.4)	
**HCV-Ab **^**b**^	Negative	2383 (98.9)	1590 (99.1)	0.775
	Positive	26 (1.1)	15 (0.9)	
**Lactate dehydrogenase (U/L) **^**c**^	/	145.20 (127.00–167.00)	145.00 (126.25–166.00)	0.504
**Alkaline phosphatase (U/L) **^**c**^	/	85.00 (71.10–100.05)	85.00 (71.00–100.30)	0.729
**De Ritis ratio **^**c**^	/	0.74 (0.56–1.13)	0.80 (0.56–1.16)	0.073
**APRI **^**c**^	/	0.07 (0.05–0.10)	0.07 (0.05–0.10)	0.318
**ALRI **^**c**^	/	9.57 (7.11–13.45)	9.42 (6.96–13.25)	0.400
**ANRI **^**c**^	/	4.77 (3.41–6.98)	4.82 (3.39–6.90)	0.996
**APPRI **^**c**^	/	0.34 (0.26–0.44)	0.34 (0.27–0.43)	0.806

*BMI* Body mass index, *De Ritis ratio* aspartate aminotransferase-to-alanine aminotransferase ratio, *APRI* aspartate aminotransferase-to-platelet ratio index, *ALRI* aspartate aminotransferase-to-lymphocyte ratio index, *ANRI* aspartate aminotransferase-to-monocyte ratio index, *APPRI* alkaline phosphatase-to-platelet ratio index

^a^Data are presented as mean (standard deviation)

^b^Data are presented as n (%)

^c^Data are presented as median (interquartile ranges)

### Determination of the optimal cut-off value

The optimal cut-off values for lactate dehydrogenase, alkaline phosphatase, De Ritis ratio, APRI, ALRI, ANRI, and APPRI were 191.00, 102.00, 1.52, 0.12, 7.50, 3.18, and 0.46, respectively (Supplementary Fig. 1). According to the optimal cut-off values of these markers, patients in the training and testing sets were divided into low- and high-level groups for further analysis.

The associations between preoperative serum liver enzyme markers and clinicopathological features were presented in Supplementary Tables 1, 2, 3, 4, 5, 6, and 7. Lactate dehydrogenase, De Ritis ratio, APRI, and APPRI were associated with age and gender; De Ritis ratio and APRI were significantly associated with tumor location, tumor diameter, and pathological classification; and only APRI, ALRI, and APPRI were associated with HBs-Ag.

### Identification of independent predictors associated with the prognosis of patients with stage II/III CRC

**Fig. 2:**
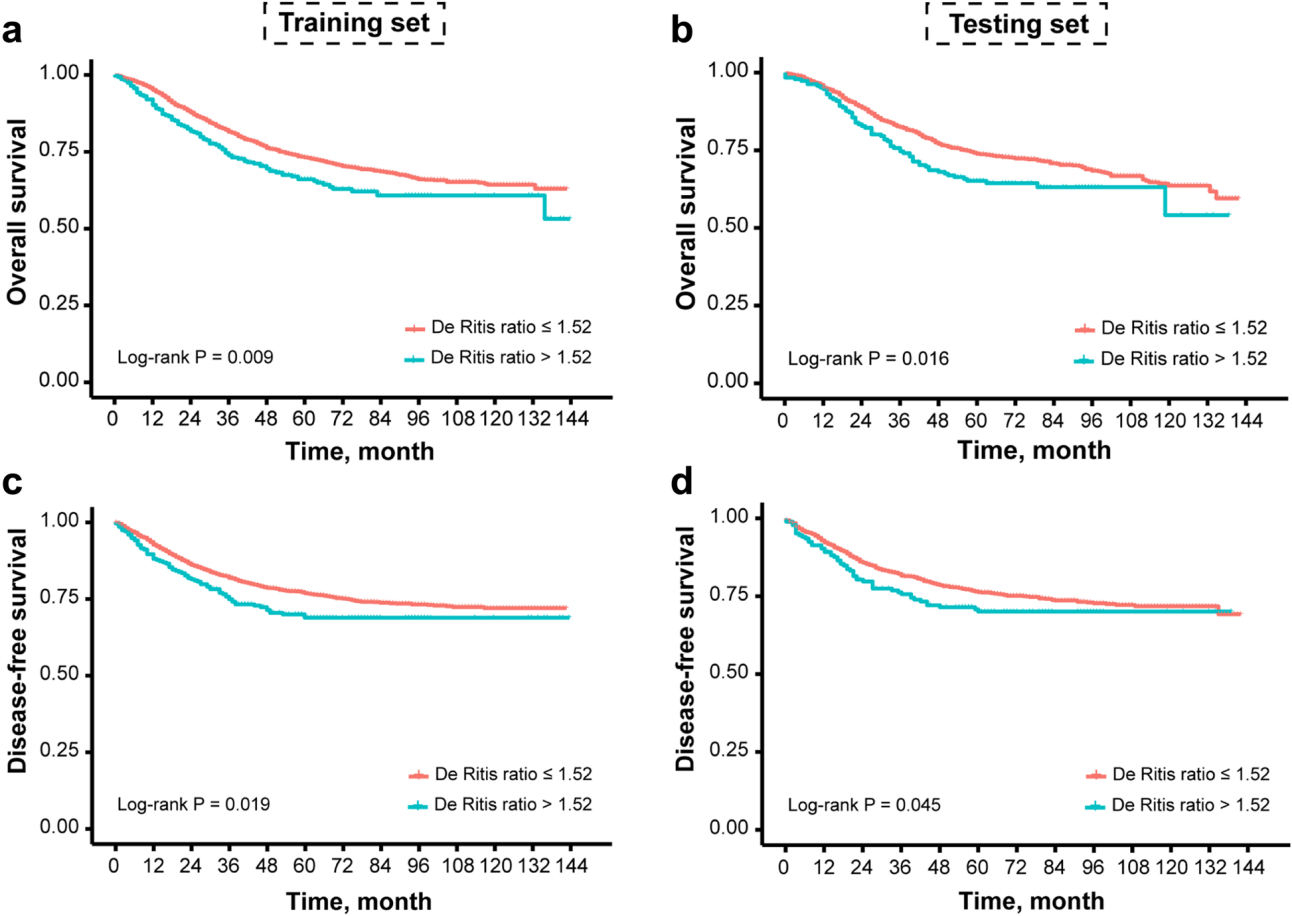
Kaplan–Meier curves of overall survival and disease-free survival in relation to De Ritis ratio. De Ritis ratio, aspartate aminotransferase-to-alanine aminotransferase ratio

In the training set, patients with a higher De Ritis ratio (> 1.52) had a significantly worse prognosis (log-rank test, *P* < 0.050) (Fig. 2a, c); so as for patients in the high-level groups of alkaline phosphatase and ALRI (Supplementary Figs. 2 and 3). The 3- and 5-year OS rates of patients in the high-level groups of alkaline phosphatase, De Ritis ratio, and ALRI were significantly lower than that of patients in the low-level groups (Supplementary Table 8). The patients in the high-level groups of alkaline phosphatase and De Ritis ratio also had a significantly poor 3- and 5-year DFS (Supplementary Table 9).

Upon multivariate Cox analysis in the training set, alkaline phosphatase (HR: 1.215, 95% CI: 1.029–1.435, *P* = 0.022), De Ritis ratio (HR: 1.269, 95% CI: 1.020–1.579, *P* = 0.033), and ALRI (HR: 1.314, 95% CI: 1.109–1.555, *P* = 0.002) were associated with the OS of patients with stage II/III CRC (Table 2), and only alkaline phosphatase (HR: 1.282, 95% CI: 1.065–1.543, *P* = 0.009) and De Ritis ratio (HR: 1.364, 95% CI: 1.068–1.743, *P* = 0.013) were associated with the DFS (Table 3).

**Table 2:** Association between predictive factors and overall survival of patients in the training and testing sets

	**Training set**				**Testing set**			
	**Univariate analysis**		**Multivariate analysis**		**Univariate analysis**		**Multivariate analysis**	
	**HR (95% CI)**	***P* value**	**HR (95% CI)**	***P* value**	**HR (95% CI)**	***P* value**	**HR (95% CI)**	***P* value**
Age (≥ 60 vs < 60)	1.702 (1.470–1.971)	< 0.001	1.510 (1.296–1.758)	** < 0.001**	1.540 (1.284–1.847)	< 0.001	1.477 (1.224–1.783)	** < 0.001**
Male (vs Female)	1.109 (0.957–1.285)	0.170	1.229 (1.057–1.427)	**0.007**	1.295 (1.074–1.563)	0.007	1.412 (1.167–1.709)	** < 0.001**
Rectum (vs Colon)	1.289 (1.115–1.490)	0.001	1.275 (1.092–1.487)	**0.002**	1.336 (1.113–1.604)	0.002	1.200 (0.991–1.452)	0.061
Tumor diameter (≥ 50 mm vs < 50 mm)	1.309 (1.122–1.528)	0.001	1.279 (1.090–1.501)	**0.003**	1.197 (0.994–1.443)	0.059	1.067 (0.879–1.295)	0.514
Pathological classification								
Infiltration or Ulceration (vs Prominence)	1.878 (1.507–2.342)	< 0.001	1.746 (1.396–2.184)	** < 0.001**	1.168 (0.869–1.571)	0.304	1.017 (0.754–1.372)	0.913
Infiltration and Ulceration (vs Prominence)	1.963 (1.677–2.298)	< 0.001	1.729 (1.473–2.029)	** < 0.001**	1.125 (0.907–1.395)	0.284	1.020 (0.818–1.271)	0.863
Differentiation degree								
Moderate (vs Well)	2.436 (1.720–3.449)	0.023	1.274 (0.923–1.759)	0.141	1.963 (1.237–3.116)	0.004	1.799 (1.131–2.861)	**0.013**
Poor (vs Well)	1.453 (1.054–2.003)	< 0.001	1.931 (1.359–2.745)	** < 0.001**	3.637 (2.226–5.943)	< 0.001	2.828 (1.723–4.641)	** < 0.001**
Mucinous adenocarcinoma or signet ring cell carcinoma (vs Adenocarcinoma)	1.162 (0.987–1.368)	0.072	1.197 (1.013–1.415)	**0.034**	1.191 (0.973–1.459)	0.091	1.112 (0.901–1.371)	0.323
TNM stage III (vs stage II)	2.335 (2.018–2.703)	< 0.001	2.201 (1.890–2.564)	** < 0.001**	2.640 (2.193–3.177)	< 0.001	2.442 (2.007–2.972)	** < 0.001**
CEA (≥ 5 ng/mL vs < 5 ng/mL)	1.528 (1.323–1.765)	< 0.001	1.231 (1.056–1.435)	**0.008**	2.006 (1.659–2.426)	< 0.001	1.537 (1.253–1.886)	** < 0.001**
CA19-9 (≥ 37U/mL vs < 37U/mL)	2.001 (1.686–2.374)	< 0.001	1.541 (1.283–1.849)	** < 0.001**	2.519 (2.049–3.097)	< 0.001	1.896 (1.512–2.377)	** < 0.001**
Postoperative chemotherapy (Yes vs No)	0.640 (0.549–0.745)	< 0.001	0.582 (0.495–0.685)	** < 0.001**	0.774 (0.644–0.931)	0.006	0.630 (0.517–0.768)	** < 0.001**
Postoperative radiotherapy (Yes vs No)	1.675 (1.260–2.227)	< 0.001	1.686 (1.254–2.268)	**0.001**	2.310 (1.636–3.261)	< 0.001	2.208 (1.542–3.162)	** < 0.001**
HBs-Ag (Positive vs Negative)	0.793 (0.550–1.143)	0.213	-	-	1.184 (0.818–1.714)	0.371	-	-
HCV-Ab (Positive vs Negative)	1.521 (0.860–2.691)	0.150	-	-	0.645 (0.207–2.006)	0.448	-	-
Lactate dehydrogenase (> 191 vs ≤ 191)	1.203 (0.956–1.513)	0.115	1.189 (0.942–1.501)	0.145	1.506 (1.142–1.987)	0.004	1.526 (1.154–2.019)	**0.003**
Alkaline phosphatase (> 102 vs ≤ 102)	1.210 (1.026–1.426)	0.023	1.215 (1.029–1.435)	**0.022**	1.244 (1.014–1.527)	0.036	1.139 (0.923–1.405)	0.227
De Ritis ratio (> 1.52 vs ≤ 1.52)	1.326 (1.072–1.640)	0.009	1.269 (1.020–1.579)	**0.033**	1.370 (1.057–1.774)	0.017	1.513 (1.160–1.973)	**0.002**
APRI (> 0.12 vs ≤ 0.12)	1.049 (0.860–1.278)	0.639	1.090 (0.890–1.335)	0.403	1.142 (0.903–1.446)	0.268	1.089 (0.858–1.381)	0.485
ALRI (> 7.50 vs ≤ 7.50)	1.306 (1.104–1.544)	0.002	1.314 (1.109–1.555)	**0.002**	1.083 (0.889–1.318)	0.428	1.114 (0.913–1.359)	0.286
ANRI (> 3.18 vs ≤ 3.18)	0.880 (0.741–1.044)	0.143	0.897 (0.753–1.069)	0.226	0.886 (0.716–1.097)	0.268	0.918 (0.737–1.142)	0.442
APPRI (> 0.46 vs ≤ 0.46)	1.075 (0.905–1.278)	0.409	1.027 (0.861–1.224)	0.769	1.162 (0.934–1.447)	0.178	1.015 (0.812–1.269)	0.898

*HR* hazard ratio, *CI* confidence interval, *De Ritis ratio* aspartate aminotransferase-to-alanine aminotransferase ratio, *APRI* aspartate aminotransferase-to-platelet ratio index, *ALRI* aspartate aminotransferase-to-lymphocyte ratio index, *ANRI* aspartate aminotransferase-to-monocyte ratio index, *APPRI* alkaline phosphatase-to-platelet ratio index

**Table 3:** Association between predictive factors and disease-free survival of patients in the training and testing sets

	**Training set**				**Testing set**			
	**Univariate analysis**		**Multivariate analysis**		**Univariate analysis**		**Multivariate analysis**	
	**HR (95% CI)**	***P* value**	**HR (95% CI)**	***P* value**	**HR (95% CI)**	***P* value**	**HR (95% CI)**	***P* value**
Age (≥ 60 vs < 60)	1.238 (1.053–1.456)	0.010	1.291 (1.090–1.528)	**0.003**	1.059 (0.870–1.290)	0.567	1.197 (0.974–1.472)	0.088
Male (vs Female)	1.083 (0.917–1.279)	0.344	1.152 (0.975–1.362)	0.098	1.274 (1.037–1.565)	0.021	1.328 (1.076–1.639)	**0.008**
Rectum (vs Colon)	1.517 (1.284–1.792)	< 0.001	1.477 (1.236–1.763)	** < 0.001**	1.852 (1.503–2.281)	< 0.001	1.642 (1.317–2.047)	** < 0.001**
Tumor diameter (≥ 50 mm vs < 50 mm)	1.265 (1.066–1.502)	0.007	1.272 (1.067–1.517)	**0.007**	1.173 (0.954–1.441)	0.129	1.129 (0.911–1.400)	0.268
Pathological classification								
Infiltration or Ulceration (vs Prominence)	1.781 (1.390–2.281)	< 0.001	1.719 (1.337–2.211)	** < 0.001**	1.299 (0.949–1.779)	0.102	1.171 (0.852–1.610)	0.330
Infiltration and Ulceration (vs Prominence)	1.779 (1.486–2.130)	< 0.001	1.611 (1.341–1.935)	** < 0.001**	1.214 (0.961–1.535)	0.104	1.079 (0.850–1.371)	0.532
Differentiation degree								
Moderate (vs Well)	0.568 (0.396–0.816)	0.002	1.017 (0.734–1.409)	0.919	0.308 (0.185–0.515)	< 0.001	1.661 (1.029–2.681)	**0.038**
Poor (vs Well)	0.654 (0.531–0.805)	< 0.001	1.379 (0.956–1.990)	0.086	0.560 (0.437–0.718)	< 0.001	2.322 (1.386–3.890)	**0.001**
Mucinous adenocarcinoma or signet ring cell carcinoma (vs Adenocarcinoma)	1.149 (0.956–1.381)	0.140	1.157 (0.958–1.397)	0.129	1.036 (0.824–1.302)	0.764	1.018 (0.803–1.291)	0.880
TNM stage III (vs stage II)	2.457 (2.083–2.898)	< 0.001	2.034 (1.713–2.415)	** < 0.001**	3.133 (2.548–3.852)	< 0.001	2.461 (1.978–3.061)	** < 0.001**
CEA (≥ 5 ng/mL vs < 5 ng/mL)	1.343 (1.141–1.581)	< 0.001	1.107 (0.929–1.319)	0.257	1.903 (1.534–2.361)	< 0.001	1.554 (1.238–1.950)	** < 0.001**
CA19-9 (≥ 37U/mL vs < 37U/mL)	1.901 (1.565–2.310)	< 0.001	1.553 (1.258–1.919)	** < 0.001**	2.227 (1.762–2.815)	< 0.001	1.820 (1.413–2.343)	** < 0.001**
Postoperative chemotherapy (Yes vs No)	1.234 (1.049–1.451)	0.011	1.094 (0.920–1.302)	0.310	1.364 (1.120–1.662)	0.002	1.103 (0.892–1.365)	0.366
Postoperative radiotherapy (Yes vs No)	2.776 (2.121–3.635)	< 0.001	2.123 (1.595–2.824)	** < 0.001**	4.256 (3.088–5.867)	< 0.001	3.008 (2.131–4.246)	** < 0.001**
HBs-Ag (Positive vs Negative)	1.006 (0.692–1.461)	0.976	-	-	1.272 (0.860–1.880)	0.228	-	-
HCV-Ab (Positive vs Negative)	0.933 (0.417–2.085)	0.866	-	-	1.266 (0.524–3.059)	0.600	-	-
Lactate dehydrogenase (> 191 vs ≤ 191)	1.172 (0.904–1.519)	0.231	1.118 (0.859–1.455)	0.408	1.343 (0.982–1.838)	0.065	1.395 (1.016–1.915)	**0.039**
Alkaline phosphatase (> 102 vs ≤ 102)	1.275 (1.061–1.532)	0.009	1.282 (1.065–1.543)	**0.009**	1.041 (0.824–1.316)	0.734	0.995 (0.783–1.265)	0.970
De Ritis ratio (> 1.52 vs ≤ 1.52)	1.327 (1.046–1.685)	0.020	1.364 (1.068–1.743)	**0.013**	1.254 (1.004–1.615)	0.045	1.470 (1.094–1.975)	**0.011**
APRI (> 0.12 vs ≤ 0.12)	1.084 (0.870–1.352)	0.473	1.077 (0.860–1.349)	0.519	1.092 (0.841–1.418)	0.510	1.058 (0.812–1.378)	0.677
ALRI (> 7.50 vs ≤ 7.50)	1.180 (0.981–1.418)	0.079	1.168 (0.970–1.406)	0.101	1.000 (0.809–1.236)	0.998	1.019 (0.823–1.263)	0.860
ANRI (> 3.18 vs ≤ 3.18)	0.844 (0.697–1.022)	0.083	0.832 (0.684–1.011)	0.065	0.773 (0.617–0.969)	0.025	0.745 (0.590–0.940)	**0.013**
APPRI (> 0.46 vs ≤ 0.46)	1.174 (0.972–1.419)	0.096	1.147 (0.946–1.390)	0.163	1.137 (0.892–1.448)	0.300	1.023 (0.799–0.310)	0.859

*HR* hazard ratio, *CI* confidence interval, *De Ritis ratio* aspartate aminotransferase-to-alanine aminotransferase ratio, *APRI* aspartate aminotransferase-to-platelet ratio index, *ALRI* aspartate aminotransferase-to-lymphocyte ratio index, *ANRI* aspartate aminotransferase-to-monocyte ratio index, *APPRI* alkaline phosphatase-to-platelet ratio index

The prognostic values of these serum liver enzyme markers were further assessed in the testing set, which was used to verify their generalizability. Patients with a high De Ritis ratio in the testing set still had significantly worse OS (Fig. 2b, Supplementary Table 10); so as for patients with high-level of alkaline phosphatase (Supplementary Fig. 4, Supplementary Table 10). The 3- and 5-year DFS rates of patients in the high-level groups of De Ritis ratio were significantly lower than that of patients in the low-level groups (Fig. 2d, Supplementary Table 11), while no statistical differences in DFS rates were observed between low and high-level groups of alkaline phosphatase (Supplementary Fig. 5, Supplementary Table 11). Based on the results of multivariate Cox analyses, only the independent prediction role of De Ritis ratio for OS (HR: 1.513, 95% CI: 1.160–1.973, *P* = 0.002) and DFS (HR: 1.470, 95% CI: 1.094–1.975, *P* = 0.011) were validated in the testing set (Tables 2 and 3).

As the sample sizes of training and testing sets were relatively small, we conducted the subgroup analyses in the entire cohort. After stratified by age, gender, tumor location, tumor diameter, CEA, CA19-9, and postoperative chemotherapy and radiotherapy, the prognostic effect of De Ritis ratio for predicting OS showed no significant difference among different subgroups (Supplementary Fig. 6a). With regard to the DFS prediction, similar results were also obtained from different subgroup analyses (Supplementary Fig. 6b).

### Development and validation of prognostic nomograms for predicting OS and DFS in patients with stage II/III CRC

**Fig. 3:**
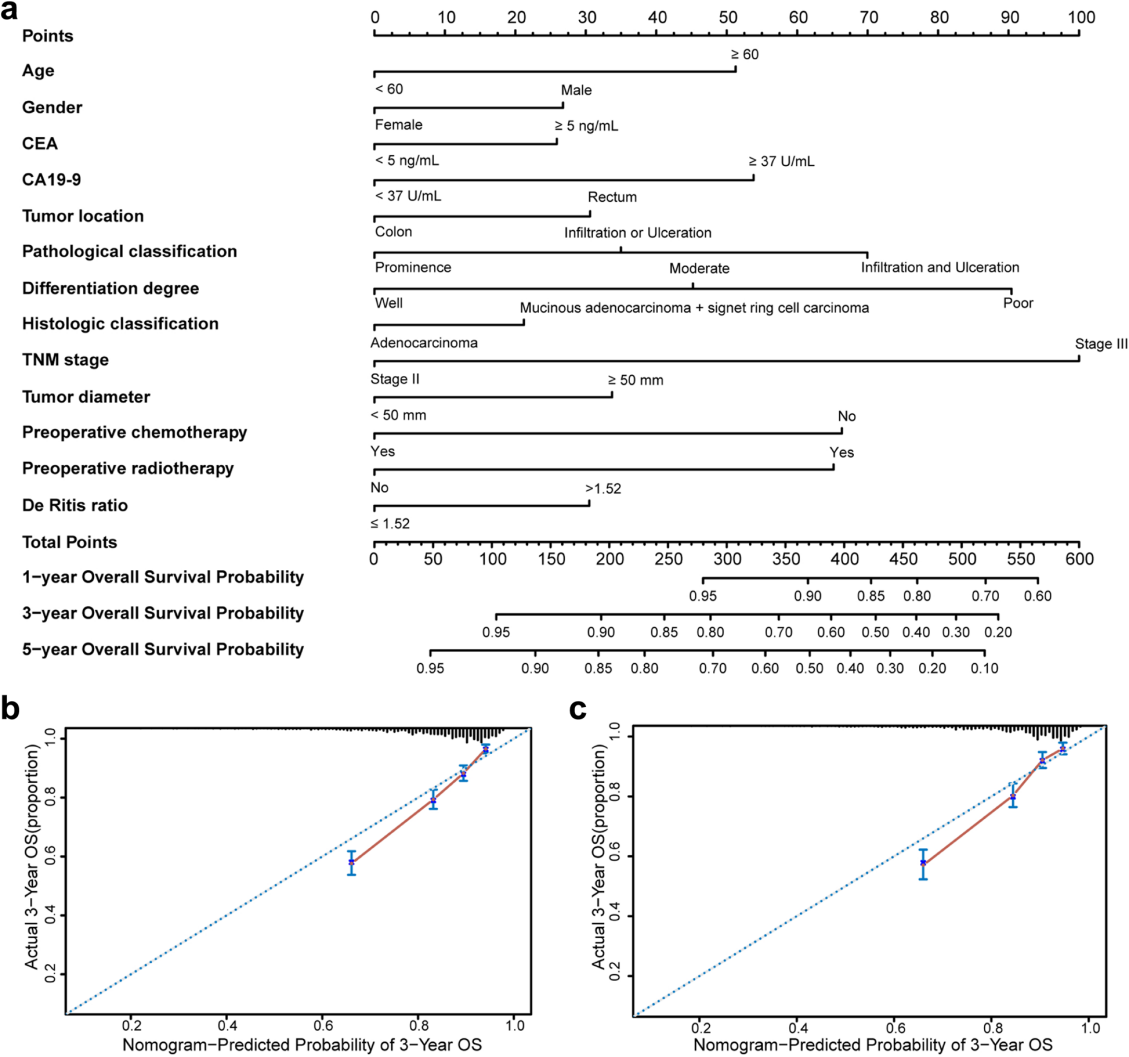
Nomograms to predict 1-, 3-, and 5-year overall survival for patients with colorectal cancer. Nomograms were performed by using significant clinicopathological features and De Ritis ratio to predict 1-, 3-, and 5-year overall survival **a** and calibration curves of the nomogram to predict overall survival at 3 years in the training set **b** and the testing set **c**

Next, nomograms that incorporated De Ritis ratio and significant clinicopathological features (age, gender, CEA, CA19-9, tumor location, pathological classification, differentiation degree, histological classification, TNM stage, tumor diameter, preoperative chemotherapy, and radiotherapy) were developed, which aimed to quantitatively predict the 1-, 3- and 5-year OS and DFS for the individual patient with stage II/III CRC (Fig. 3a, 4a). In the training set, the C-index of nomograms for predicting OS and DFS were 0.715 (95% CI: 0.697–0.733) and 0.692 (95% CI: 0.671–0.713), respectively. A similar C-index was observed when we used bootstrapping for internal validation (0.710 and 0.684) (Supplementary Table 12). The C-index of nomograms for predicting OS and DFS in the testing set were 0.730 (95% CI: 0.708–0.752) and 0.732 (95% CI: 0.707–0.756) respectively (Supplementary Table 12), which validated the good accuracy of nomograms.

**Fig. 4:**
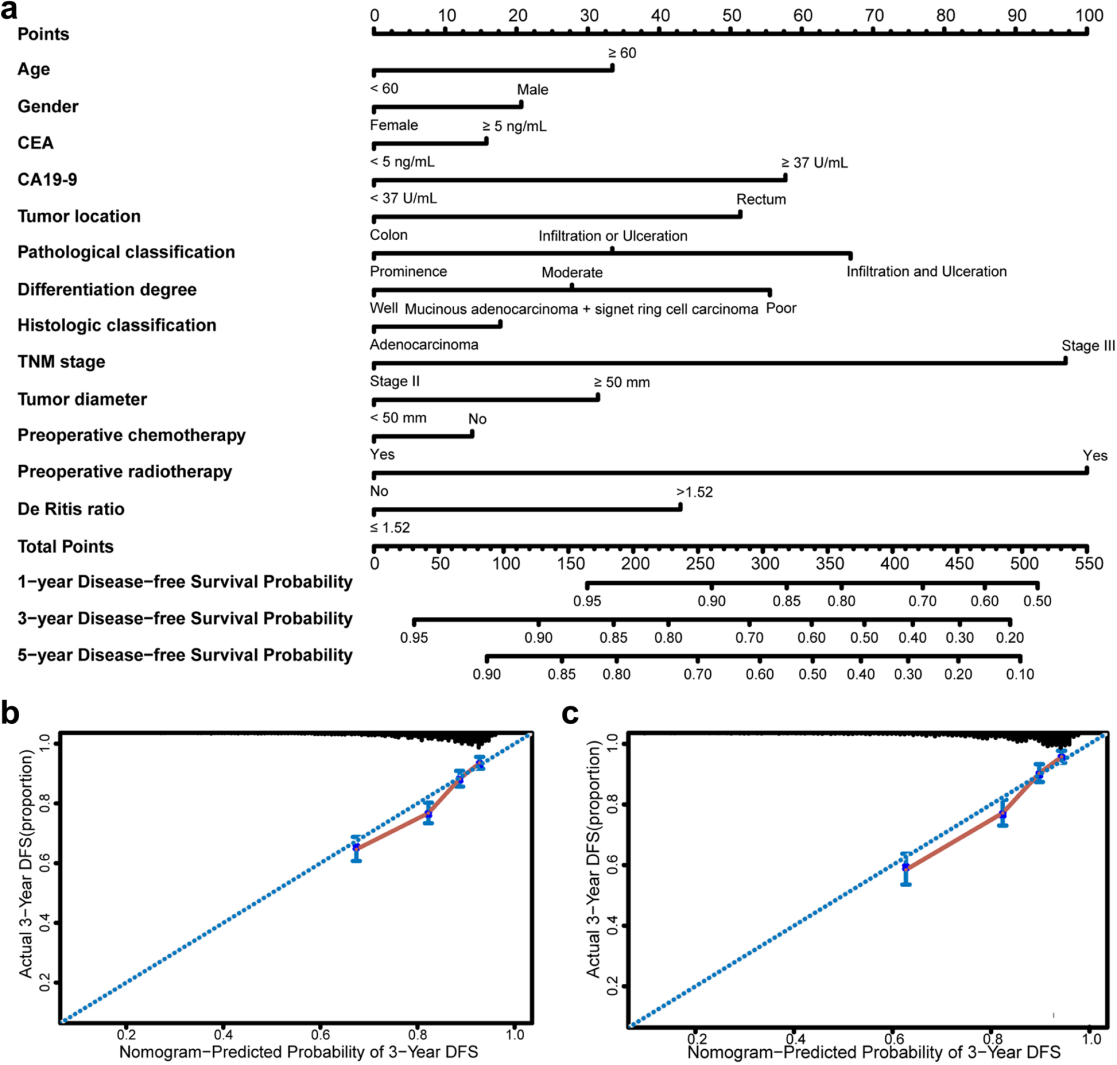
Nomograms to predict 1-, 3-, and 5-year disease-free survival for patients with colorectal cancer. Nomograms were performed by using significant clinicopathological features and De Ritis ratio to predict 1-, 3-, and 5-year disease-free survival **a** and calibration curves of the nomogram to predict disease-free survival at 3 years in the training set **b** and the testing set **c**

The calibration curves of the nomograms for predicting the probabilities of postoperative 3-year OS (Fig. 3b-c) and DFS (Fig. 4b-c) also showed well agreement between prediction and actual observation. Additionally, the C-index of the prognostic nomograms was compared to those of TNM and AJCC staging, and it was shown that the accuracy of prognostic nomograms outperformed TNM and AJCC staging both in the training and testing groups (Supplementary Table 12).

**Fig. 5:**
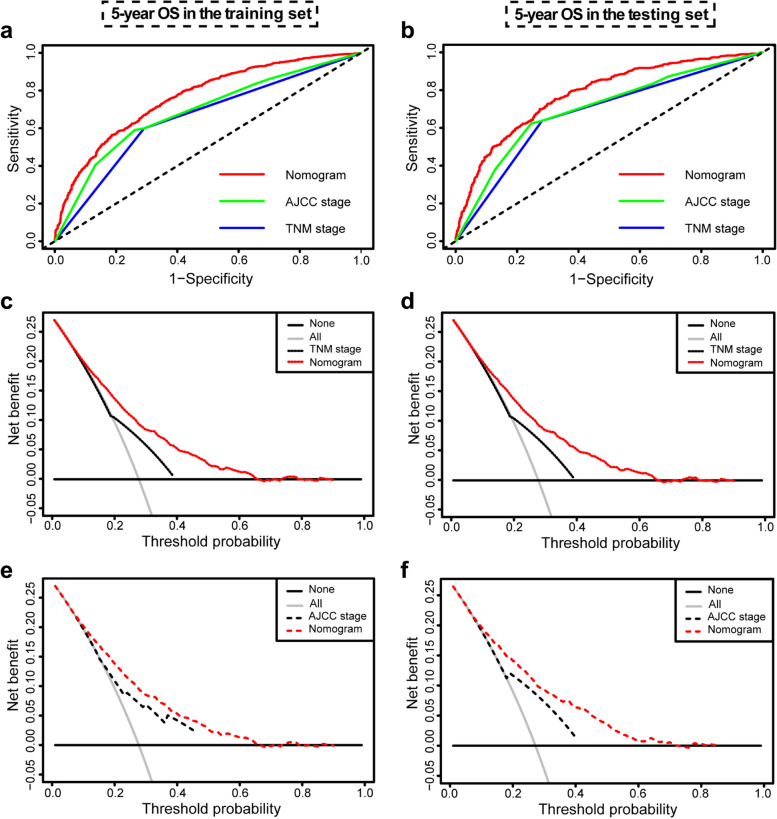
The clinical utility of the nomograms, TNM and AJCC system for predicting 5-year overall survival. AUC, area under the ROC curve. Comparisons of the time-dependent AUCs of the nomograms, TNM system, and AJCC system for 5-year overall survival prediction in the training set **a** and testing set **b**. Comparisons of the net benefits of nomograms and TNM system in the training set **c** and testing set **d**. Comparisons of the net benefits of nomograms and AJCC system in the training set **e** and testing set **f**. Black line: All patients dead. Gray line: No patients dead

**Fig. 6:**
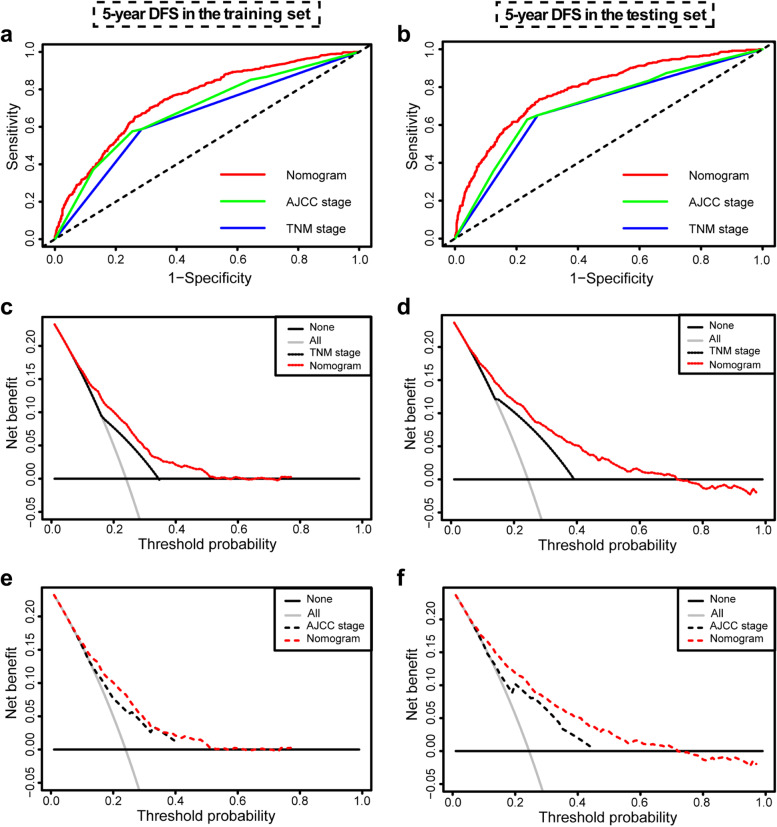
The clinical utility of the nomograms, TNM and AJCC system for predicting 5-year disease-free survival. AUC, area under the ROC curve. Comparisons of the time-dependent AUCs of the nomograms, TNM system, and AJCC system for 5-year disease-free survival prediction in the training set **a** and testing set **b**. Comparisons of the net benefits of nomograms and TNM system for 5-year disease free survival prediction in the training set **c** and testing set **d**. Comparisons of the net benefits of nomograms and AJCC system for 5-year disease-free survival prediction in the training set **e** and testing set **f**. Black line: All patients dead. Gray line: No patients dead

In the training cohort, nomograms for predicting both OS and DFS had a stable prognostic performance at various follow-up times (Supplementary Table 13), and their AUCs tended to be higher than the TNM and AJCC staging throughout the observation period, especially in 5-year OS (AUC:0.762) and DFS (AUC:0.746) prediction (Fig. 5a, 6a). The results of tests for comparing the time-dependent AUCs of nomograms with TNM and AJCC staging also showed that nomograms had better accuracy in terms of prognosis prediction (Supplementary Table 14). The time-dependent ROC curves at 5-year of the nomograms proved that the model performed well in predicting both OS (AUC:0.778) and DFS (AUC:0.787) (Fig. 5b, 6b).

### Clinical utility of the prognostic nomograms

The decision curve analyses were conducted to determine the clinical utility of the nomograms by quantifying the net benefits at different threshold probabilities. The decision curves for the nomogram in the training set indicated that when the threshold probabilities of the OS and DFS prediction were in the range of 10%-70% (Fig. 5c, e) and 10%-50% (Fig. 6c, e), respectively, a higher net clinical benefit could be achieved than the TNM staging and AJCC staging. The decision curves of the nomograms in the testing set also presented a more net benefit when the threshold probability was 10%-70% (Figs. 5d, f, 6d, f).

Collectively, the decision curve showed that the prediction ability of the nomograms was superior to the TNM and AJCC staging for patients with stage II/III CRC, which supported their favorable clinical utility in predicting OS and DFS.

## Discussion

Serum liver enzyme markers, as readily-available and non-invasive clinical parameters, have great potential for predicting the prognosis of human malignancies. Our study, for the first time, comprehensively evaluated the prognostic values of lactate dehydrogenase, alkaline phosphatase, De Ritis ratio, APRI, ALRI, ANRI, and APPRI in a large retrospective CRC cohort. Among these serum liver enzyme markers, only De Ritis ratio was identified as an independent prognostic factor for predicting the OS and DFS of patients with stage II/III CRC, which was also verified in the testing set. Based on the results of C-index, time-dependent ROC, and decision curve analyses, the nomograms combining De Ritis ratio and significant clinicopathological features had higher accuracy, improved discrimination, and greater clinical benefits in predicting the overall survival, recurrence/metastasis compared with TNM and AJCC staging.

Aspartate aminotransferase and alanine aminotransferase are enzymes produced by cancerous and non-cancerous cells, and then released into peripheral blood. Alanine aminotransferase is mainly distributed in the liver, while aspartate aminotransferase is widely expressed in different tissues including the liver, heart, skeletal muscle, and kidney [33]. Previous studies have reported serum aspartate aminotransferase as a prognostic predictor in malignant pleural mesothelioma [34], pancreatic cancer [35], and multiple myeloma [36]. These findings could be theoretically explained as, the pathological processes can lead to a higher proliferative state, tissue damage, and high tumor cell turnover, which are prone to increase the level of aspartate aminotransferase, but not alanine aminotransferase [37].

The De Ritis ratio, initially described as a characteristic of acute viral hepatitis [38], has subsequently been proposed to be a useful prognostic biomarker for predicting the survival of some other cancers, not just for liver-specific disease [11, 12]. However, to date, only Scheipner and his colleagues evaluated the prognostic role of De Ritis ratio in 536 patients with stage II/III CRC [39]. Our study retrospectively assessed and validated the prognostic value of De Ritis ratio and found that De Ritis ratio could independently predict both the OS and DFS of patients with stage II/III CRC. The prognostic value of De Ritis ratio showed no significant difference in different ages, gender, tumor location, tumor diameter, CEA, and CA19-9, which also revealed the stability effect of De Ritis ratio.

Warburg effect may explain the mechanisms of the prognostic ability of De Ritis ratio. In the view of Warburg, there was mitochondrial dysfunction in tumor cells [40]. Compared with normal cells, tumor cells rely on a greater rate of aerobic glycolysis to produce enough adenosine triphosphate to meet their proliferation and metastasis [41]. In this process, a high level of cytosolic NADH/NAD^+^ plays an essential role [42]. Aspartate aminotransferase is a component of the malate aspartate shuttle pathway, which can convert NADH/NAD^+^ to maintain enhanced glycolysis [43]. Thus, it is reasoned that the abnormal metabolism of tumor cells usually tends to increase aspartate aminotransferase level rather than alanine aminotransferase, which also supported the results that high De Ritis ratio was associated with worse OS and DFS of CRC patients.

Previous studies also indicated that lactate dehydrogenase, alkaline phosphatase, APRI, and ALRI were significant prognostic factors for metastatic CRC patients [8, 10, 17]. However, these conclusions were limited by the small sample size and no independent validation. In our study, alkaline phosphatase and ALRI were found to be associated with the OS of patients with stage II/III CRC in the training set but were not successfully verified in the testing set. Based on the analyses of existing data, our study indicated that lactate dehydrogenase, APRI, ANRI, and APPRI were not associated with the OS and DFS of stage II/III CRC patients.

Considering that individuals with hepatobiliary disorders may have abnormal levels of serum liver enzymes, which may influence the assessment of prognostic values of markers. Our study excluded CRC patients with fatty liver, cirrhosis, cholecystitis, gallstones, and gallbladder polyps. Although our study included patients with positive HBs-Ag and/or positive HCV-Ab, the prognostic values of markers were not affected due to the small proportion. Because postoperative treatment also has an important effect on prognosis, the prognostic effects of markers were adjusted by postoperative chemotherapy and radiotherapy in the multivariate Cox models.

Nomograms are widely used in oncology and have been validated to compare favorably to the conventional TNM staging systems in many cancers [44]. Our study tried to develop nomograms including preoperative serum liver enzyme markers and clinicopathological features to improve prognosis prediction of stage II/III CRC patients. The nomograms developed in the training and testing sets performed well, and their prediction accuracy was kept stable in the internal validation. Compared with TNM and AJCC systems, the nomograms held a wide range of threshold probabilities and higher net benefit, which also implied their better clinical utility.

Compared with previous studies, our study systematically investigated and validated the prognostic role of lactate dehydrogenase, alkaline phosphatase, De Ritis ratio, APRI, ALRI, ANRI, and APPRI based on a cohort containing quite a large number of patients with stage II/III CRC. In addition, new prognosis prediction models incorporating De Ritis ratio and significant clinicopathological features, also have been successfully developed. The advantages of this study include not only exploring the serum biomarkers associated with the prognosis of stage II/III CRC patients but also performing personalized survival prediction, which could help clinicians to identify patients at high risk of recurrence and death. 

Our study also has several limitations. First, all the patients in the training and testing sets came from a single-center cohort, which may bring selection bias. Multi-center cohorts should be conducted to further validate the prognostic ability of De Ritis ratio and the universal application of the optimal cut-off values of De Ritis ratio. Second, this study was a retrospective cohort and it comes with a limitation that some data on clinicopathological features are lacking, such as lymphovascular invasion, tumor budding, tumor-infiltrating lymphocyte, and microsatellite instability.

## Conclusions

Our study demonstrates that De Ritis ratio has the ability to independently predict the prognosis of patients with stage II/III CRC. The nomograms incorporating De Ritis ratio and clinicopathological features show higher accuracy, improved discrimination, and greater clinical utility in terms of personalized survival prediction.

## Supplementary Information

**Additional file 1:** **Supplementary Figure 1. **X-tile analyses of overall survival in thetraining set. **Supplementary Figure 2. **Kaplan-Meier curves and log-rank testing of overall survival in relation tolactate dehydrogenase (A), alkaline phosphatase (B), APRI (C), ALRI (D), ANRI(E), and APPRI (F) in the training set. **SupplementaryFigure 3.** Kaplan-Meier curves and log-rank testing of disease-free survivalin relation to lactate dehydrogenase (A), alkaline phosphatase (B), APRI (C),ALRI (D), ANRI (E), and APPRI (F) in the training set. **Supplementary Figure 4.** Kaplan-Meier curves and log-rank testing ofoverall survival in relation to lactate dehydrogenase (A), alkaline phosphatase(B), APRI (C), ALRI (D), ANRI (E), and APPRI (F) in the testing set. **Supplementary Figure 5.** Kaplan-Meiercurves and log-rank testing of disease-free survival in relation to lactatedehydrogenase (A), alkaline phosphatase (B), APRI (C), ALRI (D), ANRI (E), andAPPRI (F) in the testing set. **SupplementaryFigure 6.** Prognostic values of De Ritis ratio in different subgroups. Subgroupanalyses were performed in colorectal cancer patients stratified by age,gender, tumor location, tumor diameter, CEA, and CA19-9. All the analyses wereadjusted for the significant clinicopathological factors in relation to overallsurvival (A) and disease-free survival (B). **Supplementary Table 1.** Association between lactate dehydrogenase andclinicopathological features in the training set. **Supplementary Table 2.** Association between alkaline phosphatase andclinicopathological features in the training set. **Supplementary Table 3.** Association between De Ritis ratio andclinicopathological features in the training set. **Supplementary Table 4.** Association between APRI andclinicopathological features in the training set. **Supplementary Table 5.** Association between ALRI andclinicopathological features in the training set. **Supplementary Table 6.** Association between ANRI andclinicopathological features in the training set. **Supplementary Table 7.** Association between APPRI andclinicopathological features in the training set. **Supplementary Table 8.** The overall survival rates at 1-, 3-, and 5-year of patients stratified by serum liver enzyme markers in the training set. **Supplementary Table 9.** The disease-free survival rates at 1-, 3-, and 5-year of patients stratified by serum liver enzyme markers in the training set. **Supplementary Table 10. **The overall survival rates at 1-, 3-, and 5-year of patients stratified by serum liver enzyme markers in the testing set. **Supplementary Table 11. **The disease-free survival rates at 1-, 3-, and 5-year of patients stratified by serum liver enzyme markers in the testing set. **Supplementary Table 12.** The C-index of TNM staging, AJCC staging and nomograms. **SupplementaryTable 13. **Time-dependent ROC analyses for predicting overall survival and disease-free survival of patients with colorectal cancer in the training set. **Supplementary Table 14. **Time-dependent ROC analyses for predicting overall survival and disease-free survival of patients with colorectal cancer in the testing set.

## Data Availability

The datasets used and/or analysed during the current study are available from the corresponding author on reasonable request.

## Abbreviations

CRC: Colorectal cancer
TNM: Tumor, node, metastasis
De Ritis ratio: Aspartate aminotransferase-to-alanine aminotransferase ratio
AJCC: American Joint Committee on Cancer
APRI: Aspartate aminotransferase-to-platelet ratio index
ALRI: Aspartate aminotransferase-to-lymphocyte ratio index
ANRI: Aspartate aminotransferase-to-neutrophil ratio index
APPRI: Alkaline phosphatase-to-platelet ratio index
OS: Overall survival
DFS: Disease-free survival
HR: Hazard ratio
CI: Confidence interval
