# Supplementary material for: Development and validation of prognostic nomograms based on De Ritis ratio and clinicopathological features for patients with stage II/III colorectal cancer
Source: BMC Cancer. 2023 Jul 3;23:620. doi: 10.1186/s12885-023-11125-5 (PMC10318767; doi:10.1186/s12885-023-11125-5)
Supplement: Supplementary file 1 — Additional file 1: Supplementary Figure 1. X-tile analyses of overall survival in thetraining set. Supplementary Figure 2. Kaplan-Meier curves and log-rank testing of overall survival in relation tolactate dehydrogenase (A), alkaline phosphatase (B), APRI (C), ALRI (D), ANRI(E), and APPRI (F) in the training set. SupplementaryFigure 3. Kaplan-Meier curves and log-rank testing of disease-free survivalin relation to lactate dehydrogenase (A), alkaline phosphatase (B), APRI (C),ALRI (D), ANRI (E), and APPRI (F) in the training set. Supplementary Figure 4. Kaplan-Meier curves and log-rank testing ofoverall survival in relation to lactate dehydrogenase (A), alkaline phosphatase(B), APRI (C), ALRI (D), ANRI (E), and APPRI (F) in the testing set. Supplementary Figure 5. Kaplan-Meiercurves and log-rank testing of disease-free survival in relation to lactatedehydrogenase (A), alkaline phosphatase (B), APRI (C), ALRI (D), ANRI (E), andAPPRI (F) in the testing set. SupplementaryFigure 6. Prognostic values of De Ritis ratio in different subgroups. Subgroupanalyses were performed in colorectal cancer patients stratified by age,gender, tumor location, tumor diameter, CEA, and CA19-9. All the analyses wereadjusted for the significant clinicopathological factors in relation to overallsurvival (A) and disease-free survival (B). Supplementary Table 1. Association between lactate dehydrogenase andclinicopathological features in the training set. Supplementary Table 2. Association between alkaline phosphatase andclinicopathological features in the training set. Supplementary Table 3. Association between De Ritis ratio andclinicopathological features in the training set. Supplementary Table 4. Association between APRI andclinicopathological features in the training set. Supplementary Table 5. Association between ALRI andclinicopathological features in the training set. Supplementary Table 6. Association between ANRI andclinicopathological features in the training set. Supplementary Tabl [file 12885_2023_11125_MOESM1_ESM.docx]

**Supplementary Table 1.** Association between lactate dehydrogenase and clinicopathological features in the training set.

|  | **Case (%)** | **Lactate dehydrogenase** | | ***P* value** |
| --- | --- | --- | --- | --- |
|  |  | **≤191** | **>191** |  |
| **Age (year)** |  |  |  | **<0.001** |
| <60 | 1219 (50.6) | 1124 (51.9) | 95 (39.3) |  |
| ≥60 | 1190 (49.4) | 1043 (48.1) | 147 (60.7) |  |
| **Gender** |  |  |  | **<0.001** |
| Male | 1436 (59.6) | 1327 (61.2) | 109 (45.0) |  |
| Female | 973 (40.4) | 840 (38.8) | 133 (55.0) |  |
| **Tumor location** |  |  |  | 0.078 |
| Colon | 1140 (47.3) | 1012 (46.7) | 128 (52.9) |  |
| Rectum | 1269 (52.7) | 1155 (53.3) | 114 (47.1) |  |
| **Tumor diameter** |  |  |  | 0.065 |
| <50mm | 968 (40.2) | 885 (40.8) | 83 (34.3) |  |
| ≥50mm | 1441 (59.8) | 1283 (59.2) | 158 (65.7) |  |
| **Pathological classification** |  |  |  | 0.365 |
| Prominence | 1570 (65.2) | 1412 (65.2) | 158 (65.3) |  |
| Infiltration or Ulceration | 253 (10.5) | 222 (10.2) | 31 (12.8) |  |
| Infiltration and Ulceration | 586 (24.3) | 533 (24.6) | 53 (21.9) |  |
| **Differentiation degree** |  |  |  | 0.600 |
| Well | 189 (7.8) | 169 (7.8) | 20 (8.3) |  |
| Moderate | 1861 (77.3) | 1680 (77.5) | 181 (74.8) |  |
| Poor | 359 (14.9) | 318 (14.7) | 41 (16.9) |  |
| **Histologic classification** |  |  |  | 0.221 |
| Adenocarcinoma | 1819 (75.5) | 1628 (75.1) | 191 (78.9) |  |
| Mucinous adenocarcinoma or signet ring cell carcinoma | 590 (24.5) | 539 (24.9) | 51 (21.1) |  |
| **TNM staging** |  |  |  | 1.000 |
| II | 1384 (57.5) | 1245 (57.5) | 139 (57.4) |  |
| III | 1025 (42.5) | 922 (42.5) | 103 (42.6) |  |
| **Tumor invasion** |  |  |  | 0.057 |
| T1-T3 | 1061 (44.0) | 940 (43.4) | 121 (50.0) |  |
| T4 | 1348 (56.0) | 1227 (56.6) | 121 (50.0) |  |
| **CEA** |  |  |  | **0.001** |
| <5ng/mL | 1417 (58.8) | 1302 (60.1) | 115 (47.5) |  |
| ≥5ng/mL | 992 (41.2) | 865 (39.9) | 127 (52.5) |  |
| **CA19-9** |  |  |  | **<0.001** |
| <37U/mL | 2020 (83.9) | 1839 (84.9) | 181 (74.8) |  |
| ≥37U/mL | 389 (16.1) | 328 (15.1) | 61 (25.2) |  |
| **HBs-Ag** |  |  |  | 0.536 |
| Negative | 2294 (95.2) | 2066 (95.3) | 228 (94.2) |  |
| Positive | 115 (4.8) | 101 (4.7) | 14 (5.8) |  |
|  | **Case (%)** | **Lactate dehydrogenase** | | ***P* value** |
|  |  | **≤191** | **>191** |  |
| **HCV-Ab** |  |  |  | 0.466 |
| Negative | 2383 (98.9) | 2142 (98.8) | 241 (99.6) |  |
| Positive | 26 (1.1) | 25 (1.2) | 1 (0.40) |  |

**Supplementary Table 2.** Association between alkaline phosphatase and clinicopathological features in the training set.

|  | **Case (%)** | **Alkaline phosphatase** | | ***P* value** |
| --- | --- | --- | --- | --- |
|  |  | **≤102** | **>102** |  |
| **Age (year)** |  |  |  | 0.102 |
| <60 | 1219 (50.6) | 962 (51.5) | 257 (47.4) |  |
| ≥60 | 1190 (49.4) | 905 (48.5) | 285 (52.6) |  |
| **Gender** |  |  |  | 0.051 |
| Male | 1436 (59.6) | 1133 (60.7) | 303 (55.9) |  |
| Female | 973 (40.4) | 734 (39.3) | 239 (44.1) |  |
| **Tumor location** |  |  |  | 0.204 |
| Colon | 1140 (47.3) | 897 (48.0) | 243 (44.8) |  |
| Rectum | 1269 (52.7) | 970 (52.0) | 299 (55.2) |  |
| **Tumor diameter** |  |  |  | 0.769 |
| <50mm | 968 (40.2) | 753 (40.3) | 215 (39.7) |  |
| ≥50mm | 1441 (59.8) | 1114 (59.7） | 327 (60.3) |  |
| **Pathological classification** |  |  |  | 0.472 |
| Prominence | 1570 (65.2) | 1223 (65.5) | 347 (64.0) |  |
| Infiltration or Ulceration | 253 (10.5) | 200 (10.7) | 53 (9.8) |  |
| Infiltration and Ulceration | 586 (24.3) | 444 (23.8) | 142 (26.2) |  |
| **Differentiation degree** |  |  |  | 0.808 |
| Well | 189 (7.8) | 146 (7.8) | 43 (7.9) |  |
| Moderate | 1861 (77.3) | 1438 (77.0) | 423 (78.0) |  |
| Poor | 359 (14.9) | 283 (15.2) | 76 (14.0) |  |
| **Histologic classification** |  |  |  | 0.065 |
| Adenocarcinoma | 1819 (75.5) | 1393 (74.6) | 426 (78.6) |  |
| Mucinous adenocarcinoma or signet ring cell carcinoma | 590 (24.5) | 474 (25.4) | 116 (21.4) |  |
| **TNM staging** |  |  |  | 0.546 |
| II | 1384 (57.5) | 1066 (57.1) | 318 (58.7) |  |
| III | 1025 (42.5) | 801 (42.9) | 224 (41.3) |  |
| **Tumor invasion** |  |  |  | **0.037** |
| T1-T3 | 1061 (44.0) | 844 (45.2) | 217 (40.0) |  |
| T4 | 1348 (56.0) | 1023 (54.8) | 325 (60.0) |  |
| **CEA** |  |  |  | 0.075 |
| <5ng/mL | 1417 (58.8) | 1116 (59.8) | 301 (55.5) |  |
| ≥5ng/mL | 992 (41.2) | 751 (40.2) | 241 (44.5) |  |
| **CA19-9** |  |  |  | 0.698 |
| <37U/mL | 2020 (83.9) | 1570 (84.1) | 450 (83.0) |  |
| ≥37U/mL | 389 (16.1) | 297 (15.9) | 92 (17.0) |  |
| **HBs-Ag** |  |  |  | 0.407 |
| Negative | 2294 (95.2) | 1782 (95.4) | 512 (94.5) |  |
| Positive | 115 (4.8) | 85 (4.6) | 30 (5.5) |  |

|  | **Case (%)** | **Alkaline phosphatase** | | ***P* value** |
| --- | --- | --- | --- | --- |
|  |  | **≤102** | **>102** |  |

| **HCV-Ab** |  |  |  | 0.436 |
| --- | --- | --- | --- | --- |
| Negative | 2383 (98.9) | 1849 (99.0) | 534 (98.5) |  |
| Positive | 26 (1.1) | 18 (1.0) | 8 (1.5) |  |

**Supplementary Table 3.** Association between De Ritis ratio and clinicopathological features in the training set.

|  | **Case (%)** | **De Ritis ratio** | | ***P* value** |
| --- | --- | --- | --- | --- |
|  |  | **≤1.52** | **>1.52** |  |
| **Age (year)** |  |  |  | **0.014** |
| <60 | 1219 (50.6) | 1100 (51.5) | 119 (43.4) |  |
| ≥60 | 1190 (49.4) | 1035 (48.5) | 155 (56.6) |  |
| **Gender** |  |  |  | **<0.001** |
| Male | 1436 (59.6) | 1303 (61.0) | 133 (48.5) |  |
| Female | 973 (40.4) | 832 (39.0) | 141 (51.5) |  |
| **Tumor location** |  |  |  | **<0.001** |
| Colon | 1140 (47.3) | 977 (45.8) | 163 (59.5) |  |
| Rectum | 1269 (52.7) | 1158 (54.2) | 111 (40.5) |  |
| **Tumor diameter** |  |  |  | **<0.001** |
| <50mm | 968 (40.2) | 895 (41.9) | 73 (26.6) |  |
| ≥50mm | 1441 (59.8) | 1240 (58.1) | 201 (73.4) |  |
| **Pathological classification** |  |  |  | **0.027** |
| Prominence | 1570 (65.2) | 1395 (65.3) | 175 (63.9) |  |
| Infiltration or Ulceration | 253 (10.5) | 212 (10.0) | 41 (15.0) |  |
| Infiltration and Ulceration | 586 (24.3) | 528 (24.7) | 58 (21.1) |  |
| **Differentiation degree** |  |  |  | 0.787 |
| Well | 189 (7.8) | 322 (15.1) | 37 (13.5) |  |
| Moderate | 1861 (77.3) | 1646 (77.1) | 215 (78.5) |  |
| Poor | 359 (14.9) | 167 (7.8) | 22 (8.0) |  |
| **Histologic classification** |  |  |  | 0.121 |
| Adenocarcinoma | 1819 (75.5) | 1623 (76.0) | 196 (71.5) |  |
| Mucinous adenocarcinoma or signet ring cell carcinoma | 590 (24.5) | 512 (24.0) | 78 (28.5) |  |
| **TNM staging** |  |  |  | 0.523 |
| II | 1384 (57.5) | 1232 (57.7) | 152 (55.5) |  |
| III | 1025 (42.5) | 903 (42.3) | 122 (44.5) |  |
| **Tumor invasion** |  |  |  | 0.982 |
| T1-T3 | 1061 (44.0) | 941 (44.1) | 120 (43.8) |  |
| T4 | 1348 (56.0) | 1194 (55.9) | 154 (56.2) |  |
| **CEA** |  |  |  | 0.829 |
| <5ng/mL | 1417 (58.8) | 1258 (58.9) | 159 (58.0) |  |
| ≥5ng/mL | 992 (41.2) | 877 (41.1) | 115 (42.0) |  |
| **CA19-9** |  |  |  | 0.050 |
| <37U/mL | 2020 (83.9) | 1801 (84.4) | 219 (79.9) |  |
| ≥37U/mL | 389 (16.1) | 334 (15.6) | 55 (20.1) |  |
| **HBs-Ag** |  |  |  | 0.634 |
| Negative | 2294 (95.2) | 2031 (95.1) | 263 (96.0) |  |
| Positive | 115 (4.8) | 104 (4.9) | 11 (4.0) |  |
|  | **Case (%)** | **De Ritis ratio** | | ***P* value** |
|  |  | **≤1.52** | **>1.52** |  |
| **HCV-Ab** |  |  |  | 0.736 |
| Negative | 2383 (98.9) | 2113 (99.0)) | 270 (98.5) |  |
| Positive | 26 (1.1) | 22 (1.0) | 4 (1.5) |  |

**Supplementary Table 4.** Association between APRI and clinicopathological features in the training set.

|  | **Case (%)** | **APRI** | | ***P* value** |
| --- | --- | --- | --- | --- |
|  |  | **≤0.12** | **>0.12** |  |
| **Age (year)** |  |  |  | **0.013** |
| <60 | 1219 (50.6) | 1060 (51.7) | 159 (44.4) |  |
| ≥60 | 1190 (49.4) | 991 (48.3) | 199 (55.6) |  |
| **Gender** |  |  |  | **<0.001** |
| Male | 1436 (59.6) | 1169 (57.0) | 267 (74.6) |  |
| Female | 973 (40.4) | 882 (43.0) | 91 (25.4) |  |
| **Tumor location** |  |  |  | **0.009** |
| Colon | 1140 (47.3) | 994 (48.5) | 146 (40.8) |  |
| Rectum | 1269 (52.7) | 1057 (51.5) | 212 (59.2) |  |
| **Tumor diameter** |  |  |  | **0.012** |
| <50mm | 968 (40.2) | 803 (39.2) | 165 (45.8) |  |
| ≥50mm | 1441 (59.8) | 1248 (60.8) | 193 (54.2) |  |
| **Pathological classification** |  |  |  | **0.023** |
| Prominence | 1570 (65.2) | 1324 (64.6) | 246 (68.7) |  |
| Infiltration or Ulceration | 253 (10.5) | 230 (11.2) | 23 (6.4) |  |
| Infiltration and Ulceration | 586 (24.3) | 497 (24.2) | 89 (24.9) |  |
| **Differentiation degree** |  |  |  | 0.963 |
| Well | 189 (7.8) | 160 (7.8) | 29 (8.1) |  |
| Moderate | 1861 (77.3) | 1584 (77.2) | 277 (77.4) |  |
| Poor | 359 (14.9) | 307 (15.0) | 52 (14.5) |  |
| **Histologic classification** |  |  |  | **0.043** |
| Adenocarcinoma | 1819 (75.5) | 1533 (74.7) | 286 (79.9) |  |
| Mucinous adenocarcinoma or signet ring cell carcinoma | 590 (24.5) | 518 (25.3) | 72 (20.1) |  |
| **TNM staging** |  |  |  | 0.086 |
| II | 1384 (57.5) | 1163 (56.7) | 221 (61.7) |  |
| III | 1025 (42.5) | 888 (43.3) | 137 (38.3) |  |
| **Tumor invasion** |  |  |  | 0.111 |
| T1-T3 | 1061 (44.0) | 889 (43.3) | 172 (48.0) |  |
| T4 | 1348 (56.0) | 1162 (56.7) | 186 (52.0) |  |
| **CEA** |  |  |  | 0.732 |
| <5ng/mL | 1417 (58.8) | 1201 (58.6) | 216 (60.3) |  |
| ≥5ng/mL | 992 (41.2) | 850 (41.4) | 142 (39.7) |  |
| **CA19-9** |  |  |  | 0.398 |
| <37U/mL | 2020 (83.9) | 1716 (83.7) | 304 (84.9) |  |
| ≥37U/mL | 389 (16.1) | 335 (16.3) | 54 (15.1) |  |
| **HBs-Ag** |  |  |  | **<0.001** |
| Negative | 2294 (95.2) | 1973 (96.2) | 321 (89.7) |  |
| Positive | 115 (4.8) | 78 (3.8) | 37 (10.3) |  |
|  | **Case (%)** | **APRI** | | ***P* value** |
|  |  | **≤0.12** | **>0.12** |  |
| **HCV-Ab** |  |  |  | **<0.001** |
| Negative | 2383 (98.9) | 2036 (99.3) | 347 (96.9) |  |
| Positive | 26 (1.1) | 15 (0.7) | 11 (3.1) |  |

**Supplementary Table 5.** Association between ALRI and clinicopathological features in the training set.

|  | **Case (%)** | **ALRI** | | ***P* value** |
| --- | --- | --- | --- | --- |
|  |  | **≤7.50** | **>7.50** |  |
| **Age (year)** |  |  |  | 0.055 |
| <60 | 1219 (50.6) | 372 (53.8) | 847 (49.3) |  |
| ≥60 | 1190 (49.4) | 320 (46.2) | 870 (50.7) |  |
| **Gender** |  |  |  | 0.927 |
| Male | 1436 (59.6) | 411 (59.4) | 1025 (59.7) |  |
| Female | 973 (40.4) | 281 (40.6) | 692 (40.3) |  |
| **Tumor location** |  |  |  | 0.590 |
| Colon | 1140 (47.3) | 321 (46.4) | 819 (47.7) |  |
| Rectum | 1269 (52.7) | 371 (53.6) | 898 (52.3) |  |
| **Tumor diameter** |  |  |  | 0.597 |
| <50mm | 968 (40.2) | 271 (39.2) | 697 (40.6) |  |
| ≥50mm | 1441 (59.8) | 421 (60.8) | 1020 (59.4) |  |
| **Pathological classification** |  |  |  | 0.953 |
| Prominence | 1570 (65.2) | 454 (65.6) | 1116 (65.0) |  |
| Infiltration or Ulceration | 253 (10.5) | 71 (10.3) | 182 (10.6) |  |
| Infiltration and Ulceration | 586 (24.3) | 167 (24.1) | 419 (24.4) |  |
| **Differentiation degree** |  |  |  | 0.886 |
| Well | 189 (7.8) | 56 (8.1) | 133 (7.7) |  |
| Moderate | 1861 (77.3) | 530 (76.6) | 1331 (77.6) |  |
| Poor | 359 (14.9) | 106 (15.3) | 253 (14.7) |  |
| **Histologic classification** |  |  |  | **0.028** |
| Adenocarcinoma | 1819 (75.5) | 501 (72.4) | 1318 (76.8) |  |
| Mucinous adenocarcinoma or signet ring cell carcinoma | 590 (24.5) | 191 (27.6) | 399 (23.2) |  |
| **TNM staging** |  |  |  | 0.789 |
| II | 1384 (57.5) | 401 (57.9) | 983 (57.3) |  |
| III | 1025 (42.5) | 291 (42.1) | 734 (42.7) |  |
| **Tumor invasion** |  |  |  | 0.089 |
| T1-T3 | 1061 (44.0) | 324 (46.8) | 737 (42.9) |  |
| T4 | 1348 (56.0) | 368 (53.2) | 980 (57.1) |  |
| **CEA** |  |  |  | **0.017** |
| <5ng/mL | 1417 (58.8) | 432 (62.4) | 985 (57.4) |  |
| ≥5ng/mL | 992 (41.2) | 260 (37.6) | 732 (42.6) |  |
| **CA19-9** |  |  |  | 0.718 |
| <37U/mL | 2020 (83.9) | 578 (83.5) | 1442 (84.0) |  |
| ≥37U/mL | 389 (16.1) | 114 (16.5) | 275 (16.0) |  |
| **HBs-Ag** |  |  |  | **0.004** |
| Negative | 2294 (95.2) | 673 (97.3) | 1621 (94.4) |  |
| Positive | 115 (4.8) | 19 (2.7) | 96 (5.6) |  |
|  | **Case (%)** | **ALRI** | | ***P* value** |
|  |  | **≤7.50** | **>7.50** |  |
| **HCV-Ab** |  |  |  | 0.196 |
| Negative | 2383 (98.9) | 688 (99.4) | 1695 (98.7) |  |
| Positive | 26 (1.1) | 4 (0.6) | 22 (1.3) |  |

**Supplementary Table 6.** Association between ANRI and clinicopathological features in the training set.

|  | **Case (%)** | **ANRI** | | ***P* value** |
| --- | --- | --- | --- | --- |
|  |  | **≤3.18** | **>3.18** |  |
| **Age (year)** |  |  |  |  |
| <60 | 1219 (50.6) | 276 (54.0) | 943 (49.7) | 0.092 |
| ≥60 | 1190 (49.4) | 235 (46.0) | 955 (50.3) |  |
| **Gender** |  |  |  |  |
| Male | 1436 (59.6) | 303 (59.3) | 1133 (59.7) | 0.911 |
| Female | 973 (40.4) | 208 (40.7) | 765 (40.3) |  |
| **Tumor location** |  |  |  |  |
| Colon | 1140 (47.3) | 286 (56.0) | 854 (45.0) | **<0.001** |
| Rectum | 1269 (52.7) | 225 (44.0) | 1044 (55.0) |  |
| **Tumor diameter** |  |  |  |  |
| <50mm | 968 (40.2) | 145 (28.4) | 823 (43.4) | **<0.001** |
| ≥50mm | 1441 (59.8) | 366 (71.6) | 1075 (56.6) |  |
| **Pathological classification** |  |  |  |  |
| Prominence | 1570 (65.2) | 335 (65.5) | 1235 (65.1) | 0.960 |
| Infiltration or Ulceration | 253 (10.5) | 52 (10.2) | 201 (10.6) |  |
| Infiltration and Ulceration | 586 (24.3) | 124 (24.3) | 462 (24.3) |  |
| **Differentiation degree** |  |  |  |  |
| Well | 189 (7.8) | 42 (8.2) | 147 (7.7) | **0.011** |
| Moderate | 1861 (77.3) | 372 (72.8) | 1489 (78.5) |  |
| Poor | 359 (14.9) | 97 (19.0) | 262 (13.8) |  |
| **Histologic classification** |  |  |  |  |
| Adenocarcinoma | 1819 (75.5) | 357 (69.9) | 1462 (77.0) | **<0.001** |
| Mucinous adenocarcinoma or signet ring cell carcinoma | 590 (24.5) | 154 (30.1) | 436 (23.0) |  |
| **TNM staging** |  |  |  |  |
| II | 1384 (57.5) | 301 (58.9) | 1083 (57.1) | 0.485 |
| III | 1025 (42.5) | 210 (41.1) | 815 (42.9) |  |
| **Tumor invasion** |  |  |  |  |
| T1-T3 | 1061 (44.0) | 189 (37.0) | 872 (45.9) | **<0.001** |
| T4 | 1348 (56.0) | 322 (63.0) | 1026 (54.1) |  |
| **CEA** |  |  |  |  |
| <5ng/mL | 1417 (58.8) | 280 (54.8) | 1137 (59.9) | 0.050 |
| ≥5ng/mL | 992 (41.2) | 231 (45.2) | 761 (40.1) |  |
| **CA19-9** |  |  |  |  |
| <37U/mL | 2020 (83.9) | 400 (78.3) | 1620 (85.4) | **<0.001** |
| ≥37U/mL | 389 (16.1) | 111 (21.7) | 278 (14.6) |  |
| **HBs-Ag** |  |  |  |  |
| Negative | 2294 (95.2) | 495 (96.9) | 1799 (94.8) | 0.065 |
| Positive | 115 (4.8) | 16 (3.1) | 99 (5.2) |  |
|  | **Case (%)** | **ANRI** | | ***P* value** |
|  |  | **≤3.18** | **>3.18** |  |
| **HCV-Ab** |  |  |  |  |
| Negative | 2383 (98.9) | 507 (99.2) | 1876 (98.8) | 0.624 |
| Positive | 26 (1.1) | 4 (0.8) | 22 (1.2) |  |

**Supplementary Table 7.** Association between APPRI and clinicopathological features in the training set.

|  | **Case (%)** | **APPRI** | | ***P* value** |
| --- | --- | --- | --- | --- |
|  |  | **≤0.46** | **>0.46** |  |
| **Age (year)** |  |  |  | **<0.001** |
| <60 | 1219 (50.6) | 1012 (53.3) | 207 (40.4) |  |
| ≥60 | 1190 (49.4) | 885 (46.7) | 305 (59.6) |  |
| **Gender** |  |  |  | **0.008** |
| Male | 1436 (59.6) | 1104 (58.2) | 332 (64.8) |  |
| Female | 973 (40.4) | 793 (41.8) | 180 (35.2) |  |
| **Tumor location** |  |  |  | **<0.001** |
| Colon | 1140 (47.3) | 953 (50.2) | 187 (36.5) |  |
| Rectum | 1269 (52.7) | 944 (49.8) | 325 (63.5) |  |
| **Tumor diameter** |  |  |  | **0.001** |
| <50mm | 968 (40.2) | 730 (38.5) | 238 (46.5) |  |
| ≥50mm | 1441 (59.8) | 1167 (61.5) | 274 (53.5) |  |
| **Pathological classification** |  |  |  | 0.429 |
| Prominence | 1570 (65.2) | 1228 (64.7) | 342 (66.8) |  |
| Infiltration or Ulceration | 253 (10.5) | 207 (10.9) | 46 (9.0) |  |
| Infiltration and Ulceration | 586 (24.3) | 462 (24.4) | 124 (24.2) |  |
| **Differentiation degree** |  |  |  | 0.262 |
| Well | 189 (7.8) | 140 (7.4) | 49 (9.6) |  |
| Moderate | 1861 (77.3) | 1473 (77.6) | 388 (75.8) |  |
| Poor | 359 (14.9) | 284 (15.0) | 75 (14.6) |  |
| **Histologic classification** |  |  |  | **0.029** |
| Adenocarcinoma | 1819 (75.5) | 1413 (74.5) | 406 (79.3) |  |
| Mucinous adenocarcinoma or signet ring cell carcinoma | 590 (24.5) | 484 (25.5) | 106 (20.7) |  |
| **TNM staging** |  |  |  | 0.661 |
| II | 1384 (57.5) | 1085 (57.2) | 299 (58.4) |  |
| III | 1025 (42.5) | 812 (42.8) | 213 (41.6) |  |
| **Tumor invasion** |  |  |  | 0.422 |
| T1-T3 | 1061 (44.0) | 827 (43.6) | 234 (45.7) |  |
| T4 | 1348 (56.0) | 1070 (56.4) | 278 (54.3) |  |
| **CEA** |  |  |  | 0.775 |
| <5ng/mL | 1417 (58.8) | 1120 (59.0) | 297 (58.0) |  |
| ≥5ng/mL | 992 (41.2) | 777 (41.0) | 215 (42.0) |  |
| **CA19-9** |  |  |  | 0.515 |
| <37U/mL | 2020 (83.9) | 1588 (83.7) | 432 (84.4) |  |
| ≥37U/mL | 389 (16.1) | 309 (16.3) | 80 (15.6) |  |
| **HBs-Ag** |  |  |  | **<0.001** |
| Negative | 2294 (95.2) | 1825 (96.2) | 469 (91.6) |  |
| Positive | 115 (4.8) | 72 (3.8) | 43 (8.4) |  |
|  | **Case (%)** | **APPRI** | | ***P* value** |
|  |  | **≤0.46** | **>0.46** |  |
| **HCV-Ab** |  |  |  | 0.055 |
| Negative | 2383 (98.9) | 1881 (99.2) | 502 (98.0) |  |
| Positive | 26 (1.1) | 16 (0.8) | 10 (2.0) |  |

**Supplementary Table 8.** The overall survival rates at 1-, 3-, and 5-year of patients stratified by serum liver enzyme markers in the training set.

| **Group** | **1-year** | |  | **3-year** | |  | **5-year** | |
| --- | --- | --- | --- | --- | --- | --- | --- | --- |
|  | **OSR (SE)** ^a^ | ***P* value** |  | **OSR (SE)** ^a^ | ***P* value** |  | **OSR (SE)** ^a^ | ***P* value** |
| **All patients (N=2409)** | 0.945 (0.005) |  |  | 0.806 (0.008) |  |  | 0.723 (0.009) |  |
| **Lactate dehydrogenase** |  | 0.335 |  |  | **0.023** |  |  | 0.183 |
| ≤191 (N=2167) | 0.947 (0.005) |  |  | 0.812 (0.008) |  |  | 0.727 (0.010) |  |
| >191 (N=242) | 0.930 (0.016) |  |  | 0.750 (0.028) |  |  | 0.688 (0.030) |  |
| **Alkaline phosphatase** |  | 0.145 |  |  | **0.010** |  |  | **0.043** |
| ≤102 (N=1867) | 0.949 (0.005) |  |  | 0.817 (0.009) |  |  | 0.733 (0.010) |  |
| >102 (N=542) | 0.932 (0.011) |  |  | 0.766 (0.018) |  |  | 0.688 (0.020) |  |
| **De Ritis ratio** |  | **0.001** |  |  | **0.003** |  |  | **0.019** |
| ≤1.52 (N=2135) | 0.951 (0.005) |  |  | 0.814 (0.008) |  |  | 0.731 (0.010) |  |
| >1.52 (N=274) | 0.901 (0.018) |  |  | 0.738 (0.027) |  |  | 0.660 (0.029) |  |
| **APRI** |  | 0.198 |  |  | 0.661 |  |  | 0.964 |
| ≤0.12 (N=2051) | 0.942 (0.005) |  |  | 0.804 (0.009) |  |  | 0.723 (0.010) |  |
| >0.12 (N=358) | 0.961 (0.010) |  |  | 0.816 (0.021) |  |  | 0.725 (0.024) |  |
| **ALRI** |  | 0.499 |  |  | **0.012** |  |  | **0.002** |
| ≤7.50 (N=692) | 0.951 (0.008) |  |  | 0.838 (0.014) |  |  | 0.768 (0.016) |  |
| >7.50 (N=1717) | 0.943 (0.006) |  |  | 0.792 (0.010) |  |  | 0.705 (0.011) |  |
| **ANRI** |  | 0.228 |  |  | 0.107 |  |  | 0.229 |
| ≤3.18 (N=511) | 0.933 (0.011) |  |  | 0.779 (0.019) |  |  | 0.701 (0.021) |  |
| >3.18 (N=1898) | 0.948 (0.005) |  |  | 0.813 (0.009) |  |  | 0.729 (0.010) |  |
| **APPRI** |  | 0.576 |  |  | 0.750 |  |  | 1.000 |
| ≤0.46 (N=1897) | 0.943 (0.005) |  |  | 0.807 (0.009) |  |  | 0.723 (0.010) |  |
| >0.46 (N=512) | 0.951 (0.010) |  |  | 0.800 (0.018) |  |  | 0.722 (0.020) |  |

^a^ Overall survival rate (standard error).

**Supplementary Table 9.** The disease-free survival rates at 1-, 3-, and 5-year of patients stratified by serum liver enzyme markers in the training set.

| **Group** | **1-year** | |  | **3-year** | |  | **5-year** | |
| --- | --- | --- | --- | --- | --- | --- | --- | --- |
|  | **DFSR (SE)** ^a^ | ***P* value** |  | **DFSR (SE)** ^a^ | ***P* value** |  | **DFSR (SE)** ^a^ | ***P* value** |
| **All patients (N=2409)** | 0.922 (0.006) |  |  | 0.811 (0.008) |  |  | 0.760 (0.009) |  |
| **Lactate dehydrogenase** |  | 0.221 |  |  | 0.169 |  |  | 0.232 |
| ≤191 (N=2167) | 0.924 (0.006) |  |  | 0.815 (0.009) |  |  | 0.764 (0.010) |  |
| >191 (N=242) | 0.899 (0.020) |  |  | 0.775 (0.027) |  |  | 0.728 (0.030) |  |
| **Alkaline phosphatase** |  | 0.162 |  |  | **0.035** |  |  | 0.096 |
| ≤102 (N=1867) | 0.926 (0.006) |  |  | 0.821 (0.009) |  |  | 0.769 (0.010) |  |
| >102 (N=542) | 0.906 (0.013) |  |  | 0.777 (0.018) |  |  | 0.731 (0.020) |  |
| **De Ritis ratio** |  | **0.013** |  |  | **0.012** |  |  | **0.013** |
| ≤1.52 (N=2135) | 0.927 (0.006) |  |  | 0.819 (0.009) |  |  | 0.769 (0.009) |  |
| >1.52 (N=274) | 0.881 (0.020) |  |  | 0.749 (0.027) |  |  | 0.689 (0.030) |  |
| **APRI** |  | 0.854 |  |  | 0.749 |  |  | 0.847 |
| ≤0.12 (N=2051) | 0.922 (0.006) |  |  | 0.809 (0.009) |  |  | 0.761 (0.010) |  |
| >0.12 (N=358) | 0.918 (0.015) |  |  | 0.820 (0.021) |  |  | 0.756 (0.024) |  |
| **ALRI** |  | 0.875 |  |  | 0.268 |  |  | 0.098 |
| ≤7.50 (N=692) | 0.924 (0.010) |  |  | 0.828 (0.015) |  |  | 0.788 (0.016) |  |
| >7.50 (N=1717) | 0.921 (0.007) |  |  | 0.804 (0.010) |  |  | 0.748 (0.011) |  |
| **ANRI** |  | 0.188 |  |  | **0.036** |  |  | 0.070 |
| ≤3.18 (N=511) | 0.906 (0.013) |  |  | 0.773 (0.019) |  |  | 0.724 (0.021) |  |
| >3.18 (N=1898) | 0.926 (0.006) |  |  | 0.821 (0.009) |  |  | 0.769 (0.010) |  |
| **APPRI** |  | 0.857 |  |  | 0.791 |  |  | 0.343 |
| ≤0.46 (N=1897) | 0.923 (0.006) |  |  | 0.812 (0.009) |  |  | 0.765 (0.010) |  |
| >0.46 (N=512) | 0.919 (0.012) |  |  | 0.807 (0.018) |  |  | 0.743 (0.020) |  |

^a^ Disease-free survival rate (standard error).

**Supplementary Table 10.** The overall survival rates at 1-, 3-, and 5-year of patients stratified by serum liver enzyme markers in the testing set.

| **Group** | **1-year** | |  | **3-year** | |  | **5-year** | |
| --- | --- | --- | --- | --- | --- | --- | --- | --- |
|  | **OSR (SE)** ^a^ | ***P* value** |  | **OSR (SE)** ^a^ | ***P* value** |  | **OSR (SE)** ^a^ | ***P* value** |
| **All patients (N=1605)** | 0.950 (0.005) |  |  | 0.815 (0.010) |  |  | 0.728 (0.011) |  |
| **Lactate dehydrogenase** |  | **0.022** |  |  | 0.136 |  |  | **0.041** |
| ≤191 (N=1452) | 0.955 (0.005) |  |  | 0.820 (0.010) |  |  | 0.737 (0.012) |  |
| >191 (N=153) | 0.908 (0.024) |  |  | 0.765 (0.035) |  |  | 0.646 (0.040) |  |
| **Alkaline phosphatase** |  | 0.366 |  |  | 0.057 |  |  | **0.028** |
| ≤102 (N=1240) | 0.953 (0.006) |  |  | 0.826 (0.011) |  |  | 0.742 (0.013) |  |
| >102 (N=365) | 0.940 (0.012) |  |  | 0.780 (0.022) |  |  | 0.681 (0.025) |  |
| **De Ritis ratio** |  | 0.935 |  |  | **0.010** |  |  | **0.008** |
| ≤1.52 (N=1419) | 0.951 (0.006) |  |  | 0.824 (0.010) |  |  | 0.738 (0.012) |  |
| >1.52 (N=186) | 0.946 (0.017) |  |  | 0.746 (0.032) |  |  | 0.651 (0.035) |  |
| **APRI** |  | 0.444 |  |  | 0.142 |  |  | 0.307 |
| ≤0.12 (N=1345) | 0.948 (0.006) |  |  | 0.822 (0.011) |  |  | 0.733 (0.012) |  |
| >0.12 (N=260) | 0.962 (0.012) |  |  | 0.781 (0.026) |  |  | 0.702 (0.029) |  |
| **ALRI** |  | 0.426 |  |  | 0.826 |  |  | 1.000 |
| ≤7.50 (N=507) | 0.943 (0.010) |  |  | 0.812 (0.017) |  |  | 0.730 (0.020) |  |
| >7.50 (N=1098) | 0.953 (0.006) |  |  | 0.817 (0.012) |  |  | 0.727 (0.014) |  |
| **ANRI** |  | 0.356 |  |  | 0.210 |  |  | 0.192 |
| ≤3.18 (N=345) | 0.939 (0.013) |  |  | 0.791 (0.022) |  |  | 0.701 (0.025) |  |
| >3.18 (N=1260) | 0.953 (0.006) |  |  | 0.822 (0.011) |  |  | 0.736 (0.013) |  |
| **APPRI** |  | 0.143 |  |  | 0.631 |  |  | 0.248 |
| ≤0.46 (N=1293) | 0.946 (0.006) |  |  | 0.818 (0.011) |  |  | 0.736 (0.013) |  |
| >0.46 (N=312) | 0.968 (0.010) |  |  | 0.803 (0.023) |  |  | 0.696 (0.027) |  |

^a^ Overall survival rate (standard error).

**Supplementary Table 11.** The disease-free survival rates at 1-, 3-, and 5-year of patients stratified by serum liver enzyme markers in the testing set.

| **Group** | **1-year** | |  | **3-year** | |  | **5-year** | |
| --- | --- | --- | --- | --- | --- | --- | --- | --- |
|  | **DFSR (SE)** ^a^ | ***P* value** |  | **DFSR (SE)** ^a^ | ***P* value** |  | **DFSR (SE)** ^a^ | ***P* value** |
| **All patients (N=1605)** | 0.919 (0.007) |  |  | 0.809 (0.010) |  |  | 0.756 (0.011) |  |
| **Lactate dehydrogenase** |  | 0.104 |  |  | 0.238 |  |  | 0.128 |
| ≤191 (N=1452) | 0.923 (0.007) |  |  | 0.813 (0.010) |  |  | 0.762 (0.012) |  |
| >191 (N=153) | 0.879 (0.027) |  |  | 0.764 (0.035) |  |  | 0.691 (0.040) |  |
| **Alkaline phosphatase** |  | 1.000 |  |  | 0.497 |  |  | 0.883 |
| ≤102 (N=1240) | 0.919 (0.008) |  |  | 0.813 (0.011) |  |  | 0.758 (0.013) |  |
| >102 (N=365) | 0.920 (0.014) |  |  | 0.793 (0.022) |  |  | 0.748 (0.024) |  |
| **De Ritis ratio** |  | 0.192 |  |  | **0.041** |  |  | **0.044** |
| ≤1.52 (N=1419) | 0.922 (0.007) |  |  | 0.816 (0.011) |  |  | 0.763 (0.012) |  |
| >1.52 (N=186) | 0.892 (0.023) |  |  | 0.756 (0.032) |  |  | 0.700 (0.035) |  |
| **APRI** |  | 0.273 |  |  | 0.635 |  |  | 0.741 |
| ≤0.12 (N=1345) | 0.915 (0.008) |  |  | 0.812 (0.011) |  |  | 0.758 (0.012) |  |
| >0.12 (N=260) | 0.938 (0.015) |  |  | 0.791 (0.026) |  |  | 0.740 (0.029) |  |
| **ALRI** |  | 0.587 |  |  | 0.550 |  |  | 0.559 |
| ≤7.50 (N=507) | 0.913 (0.013) |  |  | 0.800 (0.018) |  |  | 0.747 (0.020) |  |
| >7.50 (N=1098) | 0.922 (0.008) |  |  | 0.813 (0.012) |  |  | 0.760 (0.013) |  |
| **ANRI** |  | 0.197 |  |  | **0.012** |  |  | **0.035** |
| ≤3.18 (N=345) | 0.901 (0.016) |  |  | 0.759 (0.024) |  |  | 0.712 (0.025) |  |
| >3.18 (N=1260) | 0.924 (0.008) |  |  | 0.822 (0.011) |  |  | 0.768 (0.012) |  |
| **APPRI** |  | 0.741 |  |  | 0.136 |  |  | 0.439 |
| ≤0.46 (N=1293) | 0.920 (0.008) |  |  | 0.816 (0.011) |  |  | 0.760 (0.012) |  |
| >0.46 (N=312) | 0.913 (0.016) |  |  | 0.776 (0.024) |  |  | 0.737 (0.026) |  |

^a^ Disease-free survival rate (standard error).

**Supplementary Table 12.** The C-index of TNM staging, AJCC staging and nomograms.

| **Models** | **Training set (n=2409)** | |  | **Testing set (n=1605)** | |
| --- | --- | --- | --- | --- | --- |
|  | **C-index** | **95% CI** |  | **C-index** | **95% CI** |
| **Overall Survival** | | | | | |
| TNM staging | 0.615 | 0.598-0.633 |  | 0.630^*^ | 0.609-0.652 |
| AJCC staging | 0.651 | 0.632-0.671 |  | 0.662^*^ | 0.637-0.686 |
| Nomogram | 0.715 | 0.697-0.733 |  | 0.730^*^ | 0.708-0.752 |
| **Disease-free Survival** | | | | | |
| TNM staging | 0.613 | 0.593-0.633 |  | 0.639^*^ | 0.615-0.662 |
| AJCC staging | 0.652 | 0.630-0.673 |  | 0.663^*^ | 0.637-0.690 |
| Nomogram | 0.692 | 0.671-0.713 |  | 0.732^*^ | 0.707-0.756 |

**P*>0.05. The C-index of TNM staging, AJCC staging and nomogram show no statistical differences between training and testing sets.

**Supplementary Table 13.** Time-dependent ROC analyses for predicting overall survival and disease-free survival of patients with colorectal cancer in the training set.

| **AUCs** | **Overall survival** | | | | |  | **Disease-free survival** | | | | |
| --- | --- | --- | --- | --- | --- | --- | --- | --- | --- | --- | --- |
|  | **Nomogram** | **TNM stage** | **AJCC stage** | ***P* value^a^** | ***P* value^b^** |  | **Nomogram** | **TNM stage** | **AJCC stage** | ***P* value^a^** | ***P* value^b^** |
| 1-year | 0.791 | 0.677 | 0.735 | <0.001 | 0.043 |  | 0.735 | 0.660 | 0.725 | <0.001 | 0.993 |
| 2-year | 0.776 | 0.679 | 0.733 | <0.001 | 0.018 |  | 0.737 | 0.654 | 0.709 | <0.001 | 0.159 |
| 3-year | 0.776 | 0.670 | 0.715 | <0.001 | <0.001 |  | 0.732 | 0.643 | 0.694 | <0.001 | 0.008 |
| 4-year | 0.770 | 0.657 | 0.692 | <0.001 | <0.001 |  | 0.736 | 0.644 | 0.684 | <0.001 | <0.001 |
| **5-year** | **0.762** | **0.654** | **0.687** | **<0.001** | **<0.001** |  | **0.746** | **0.651** | **0.689** | **<0.001** | **<0.001** |
| 6-year | 0.770 | 0.667 | 0.684 | <0.001 | <0.001 |  | 0.758 | 0.670 | 0.689 | <0.001 | <0.001 |
| 7-year | 0.779 | 0.690 | 0.701 | <0.001 | <0.001 |  | 0.770 | 0.700 | 0.712 | <0.001 | <0.001 |
| 8-year | 0.787 | 0.702 | 0.700 | <0.001 | <0.001 |  | 0.793 | 0.721 | 0.724 | <0.001 | <0.001 |
| 9-year | 0.777 | 0.710 | 0.708 | <0.001 | 0.001 |  | 0.797 | 0.738 | 0.738 | <0.001 | 0.001 |
| 10-year | 0.782 | 0.704 | 0.705 | <0.001 | 0.003 |  | 0.800 | 0.737 | 0.736 | <0.001 | 0.004 |
| 11-year | 0.817 | 0.725 | 0.786 | 0.001 | 0.874 |  | 0.839 | 0.762 | 0.810 | 0.002 | 0.764 |

ROC, receiver operating characteristic; AUC, area under the ROC curve.

^a^ Comparison of the AUCs of nomogram and TNM stage.

^b^ Comparison of the AUCs of nomogram and AJCC stage.

**Supplementary Table 14.** Time-dependent ROC analyses for predicting overall survival and disease-free survival of patients with colorectal cancer in the testing set.

| **AUCs** | **Overall survival** | | | | |  | **Disease-free survival** | | | | |
| --- | --- | --- | --- | --- | --- | --- | --- | --- | --- | --- | --- |
|  | **Nomogram** | **TNM stage** | **AJCC stage** | ***P* value^a^** | ***P* value^b^** |  | **Nomogram** | **TNM stage** | **AJCC stage** | ***P* value^a^** | ***P* value^b^** |
| 1-year | 0.814 | 0.659 | 0.714 | <0.001 | 0.011 |  | 0.775 | 0.651 | 0.687 | <0.001 | 0.002 |
| 2-year | 0.771 | 0.669 | 0.712 | <0.001 | 0.015 |  | 0.756 | 0.658 | 0.697 | <0.001 | 0.003 |
| 3-year | 0.783 | 0.687 | 0.727 | <0.001 | 0.003 |  | 0.774 | 0.680 | 0.716 | <0.001 | <0.001 |
| 4-year | 0.780 | 0.681 | 0.711 | <0.001 | <0.001 |  | 0.782 | 0.685 | 0.707 | <0.001 | <0.001 |
| **5-year** | **0.778** | **0.678** | **0.702** | **<0.001** | **<0.001** |  | **0.787** | **0.691** | **0.708** | **<0.001** | **<0.001** |
| 6-year | 0.778 | 0.678 | 0.689 | <0.001 | <0.001 |  | 0.781 | 0.695 | 0.702 | <0.001 | <0.001 |
| 7-year | 0.783 | 0.702 | 0.703 | <0.001 | <0.001 |  | 0.805 | 0.731 | 0.728 | <0.001 | <0.001 |
| 8-year | 0.797 | 0.710 | 0.710 | <0.001 | <0.001 |  | 0.818 | 0.744 | 0.738 | <0.001 | <0.001 |
| 9-year | 0.810 | 0.725 | 0.736 | <0.001 | 0.003 |  | 0.838 | 0.772 | 0.783 | <0.001 | 0.010 |
| 10-year | 0.813 | 0.730 | 0.748 | 0.002 | 0.061 |  | 0.843 | 0.789 | 0.807 | 0.011 | 0.330 |
| 11-year | 0.779 | 0.733 | 0.801 | 0.693 | 0.994 |  | 0.834 | 0.796 | 0.856 | 0.479 | 0.937 |

ROC, receiver operating characteristic; AUC, area under the ROC curve.

^a^ Comparison of the AUCs of nomogram and TNM stage.

^b^ Comparison of the AUCs of nomogram and AJCC stage.

**Supplementary Figure 1.** X-tile analyses of overall survival in the training set.

(A) Lactate dehydrogenase. (B) Alkaline phosphatase. (C) De Ritis ratio. (D) APRI. (E) ALRI. (F) ANRI. (G) APPRI.

**
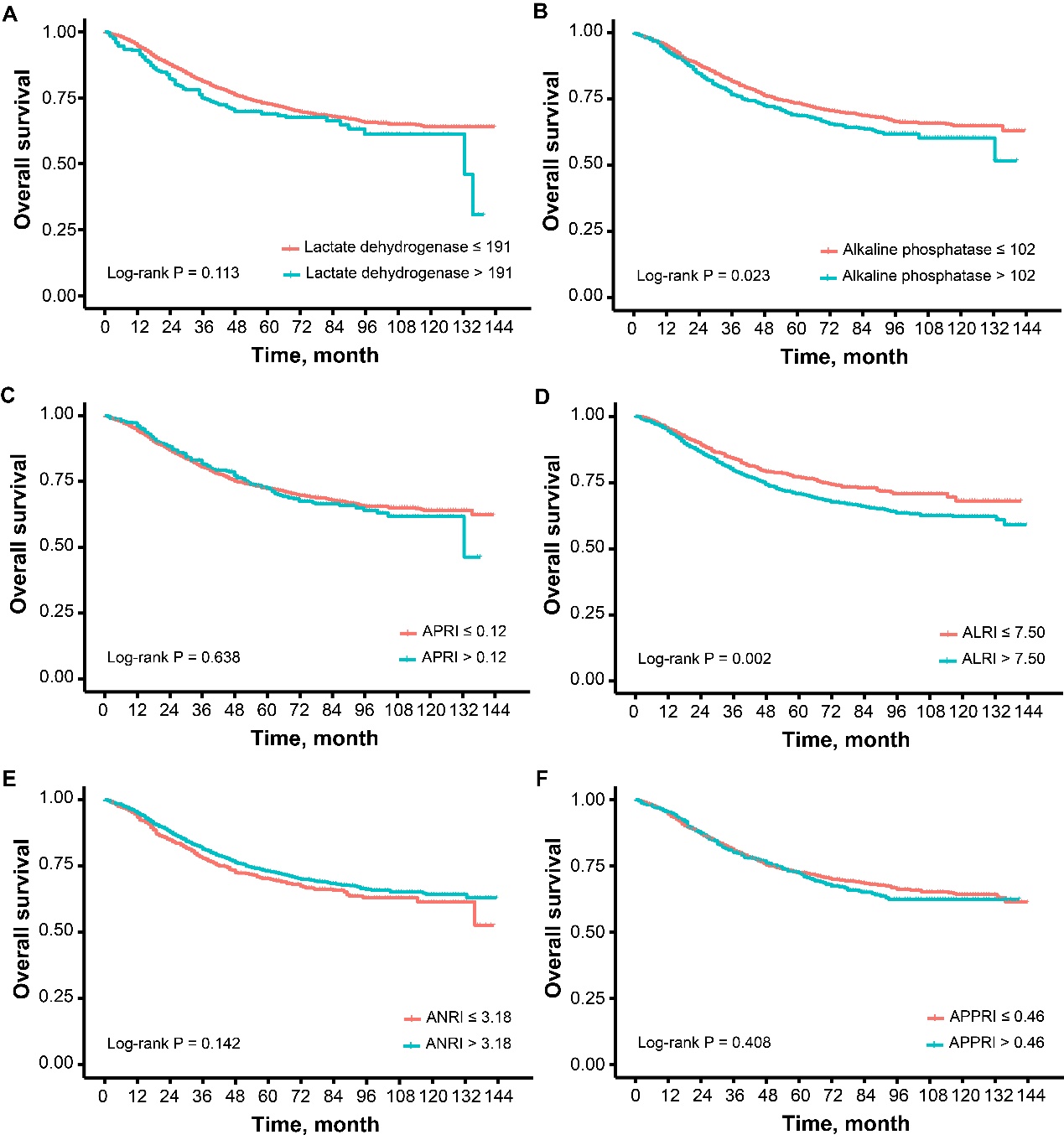
**

**Supplementary Figure 2.** Kaplan-Meier curves and log-rank testing of overall survival in relation to lactate dehydrogenase (A), alkaline phosphatase (B), APRI (C), ALRI (D), ANRI (E), and APPRI (F) in the training set.


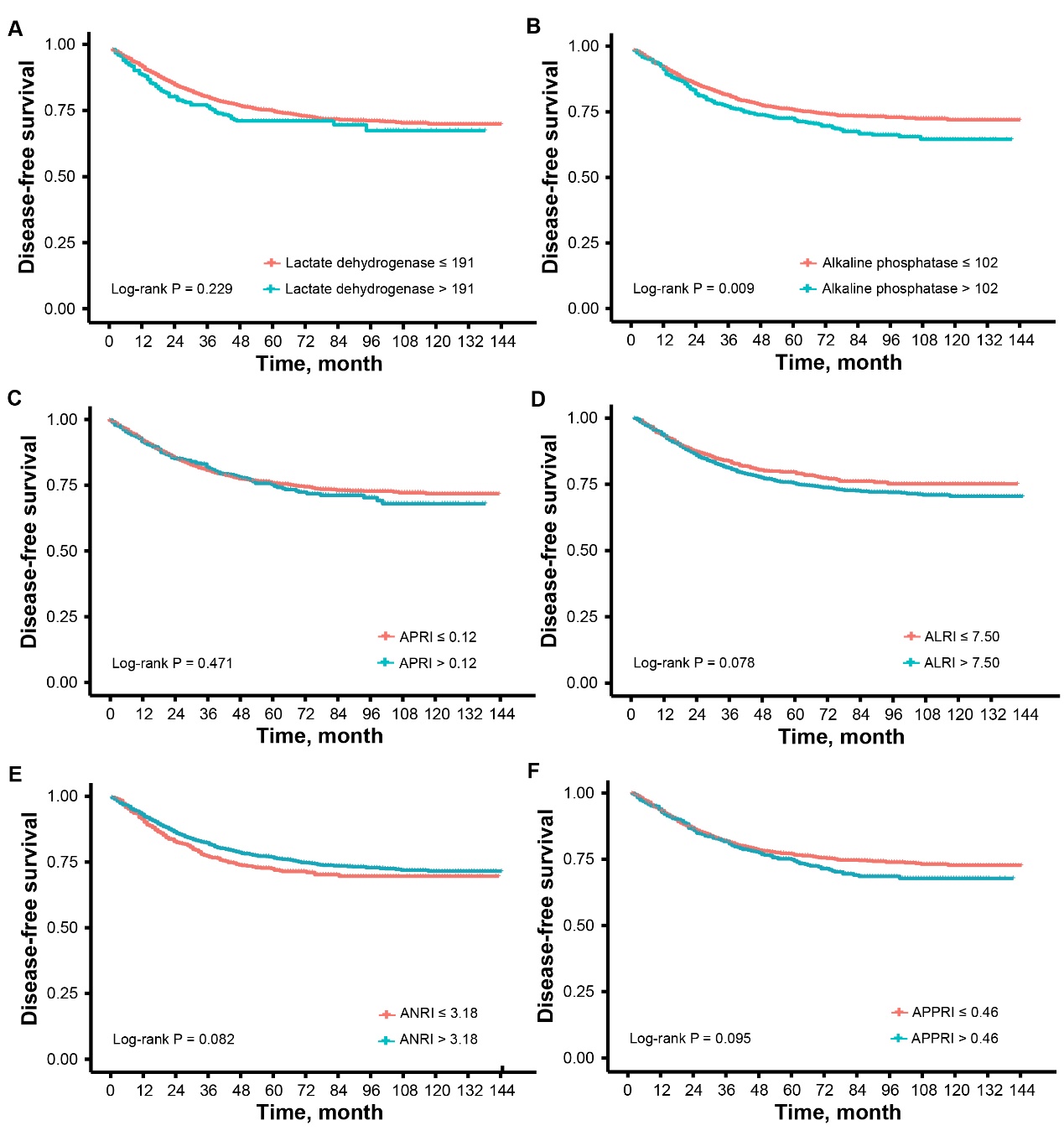


**Supplementary Figure 3.** Kaplan-Meier curves and log-rank testing of disease-free survival in relation to lactate dehydrogenase (A), alkaline phosphatase (B), APRI (C), ALRI (D), ANRI (E), and APPRI (F) in the training set.

**
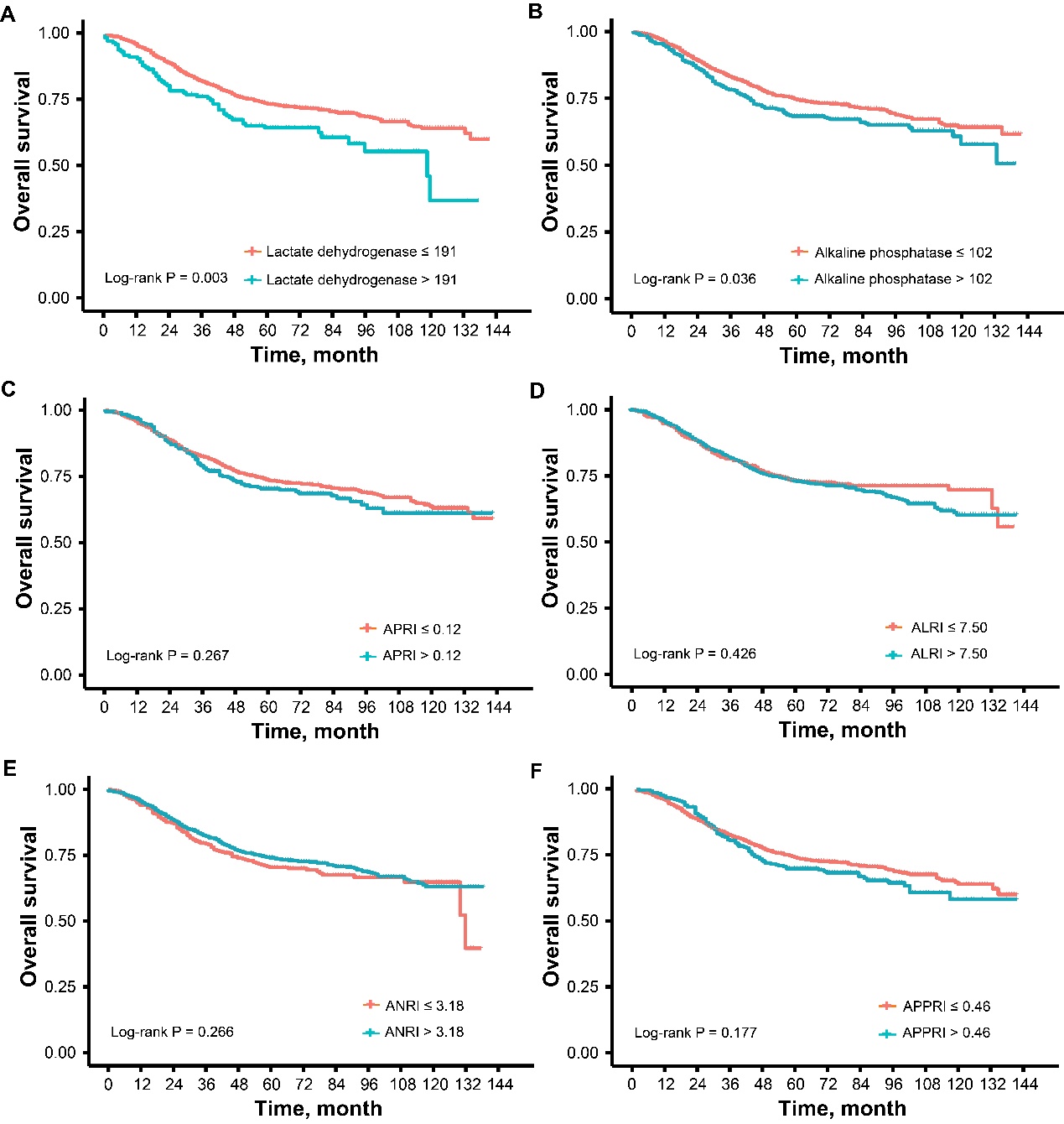
**

**Supplementary Figure 4.** Kaplan-Meier curves and log-rank testing of overall survival in relation to lactate dehydrogenase (A), alkaline phosphatase (B), APRI (C), ALRI (D), ANRI (E), and APPRI (F) in the testing set.


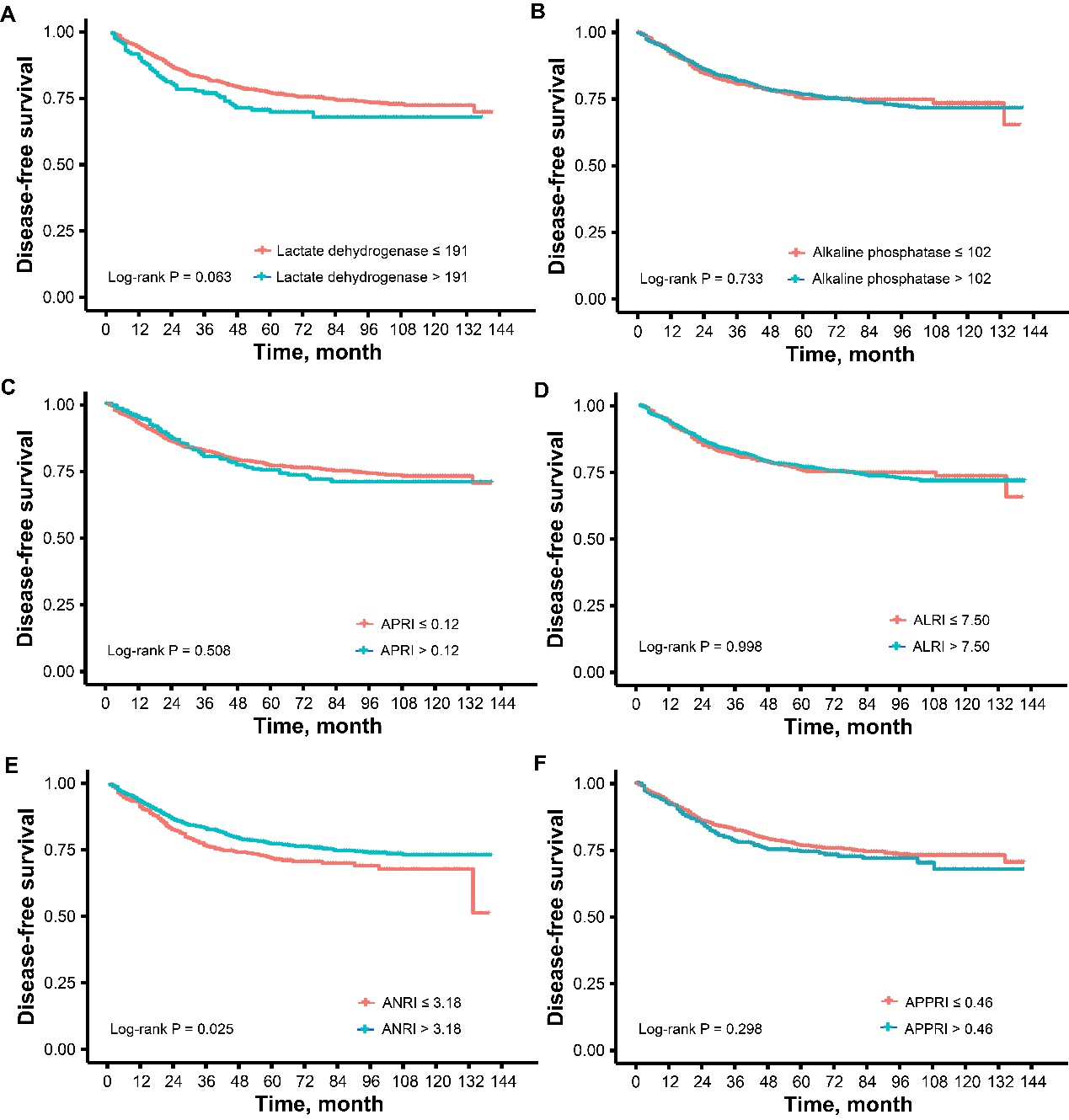


**Supplementary Figure 5.** Kaplan-Meier curves and log-rank testing of disease-free survival in relation to lactate dehydrogenase (A), alkaline phosphatase (B), APRI (C), ALRI (D), ANRI (E), and APPRI (F) in the testing set.


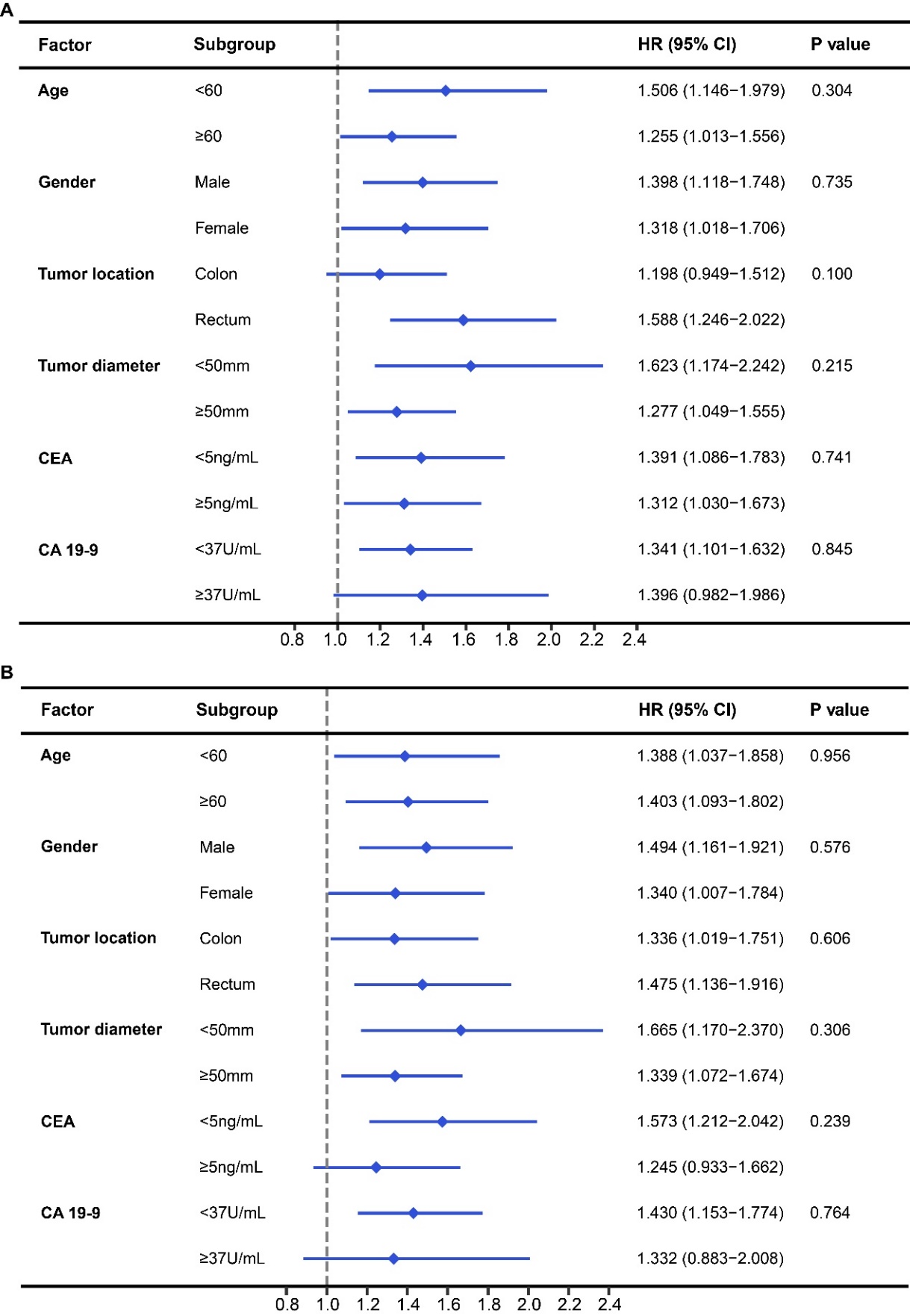


**Supplementary Figure 6.** Prognostic values of De Ritis ratio in different subgroups

Subgroup analyses were performed in colorectal cancer patients stratified by age, gender, tumor location, tumor diameter, CEA, and CA19-9. All the analyses were adjusted for the significant clinicopathological factors in relation to overall survival (A) and disease-free survival (B).
